# Supplementary material for: Not a Benign (Mis)Label: Penicillin Allergy Education for the Nonallergist
Source: MedEdPORTAL. 2024 Sep 27;20:11440. doi: 10.15766/mep_2374-8265.11440 (PMC11427523; doi:10.15766/mep_2374-8265.11440)
Supplement: Supplementary file 1 — PenEd Facilitator Guide.docxPenEd Editable Survey With Answers.docxPenEd PowerPoint.pptxPenEd Student Scripts for Role-Play.docx [file mep_2374-8265.11440-s001.zip › B. PenEd Editable Survey With Answers.docx]

Name: ________________________________

Is this a pre-test or a post-test? (Choose pre-test if you have not yet taken the course and choose post-test if you have completed the course)

1. Pre-test
2. Post-test

Rate as important the degree to which you agree with the following:

|  | Strongly disagree | Disagree | Neither agree nor disagree | Agree | Strongly agree |
| --- | --- | --- | --- | --- | --- |
| I feel prepared to prescribed antibiotics for the penicillin allergic | 1 | 2 | 3 | 4 | 5 |
| I feel prepared to determine if a patient has a history of an allergic reaction that was severe or life threatening | 1 | 2 | 3 | 4 | 5 |
| Having a penicillin allergy label has important personal health and public health consequences | 1 | 2 | 3 | 4 | 5 |

1. Once a patient develops an allergy to penicillin they will always have this allergy.

a. True

b. False

2. Skin testing is a valid tool for assessing whether a patient is penicillin-allergic.

a. True

b. False

3. Over 90% of individuals with a penicillin allergy can safely take penicillin.

a. True

b. False

4. A family history of drug allergy is a risk factor for penicillin allergy.

a. True

b. False

5. Patients with penicillin reaction histories such as headache or isolated gastrointestinal symptoms require further testing before they can safely take penicillin.

a. True

b. False

6. Patients with a history of penicillin allergy label should not be referred for drug allergy evaluation until they need antibiotics.

a. True

b. False

7. Figuring out whether a patient is truly allergic to penicillin (also known as “delabeling”) is a cost-saving intervention.

a. True

b. False

8. Skin testing should be used for patients with a penicillin allergy history of anaphylaxis or recent reaction that is suspected to be IgE-mediated.

a. True

b. False

9. Severe cutaneous adverse reaction (e.g. Drug reaction with eosinophilia and systemic symptoms (DRESS), Stevens Johnson Syndrome (SJS), Toxic Epidermal Necrolysis (TEN)) to a beta-lactam is considered an absolute contraindication to receiving any beta-lactam again, even by a process of desensitization.

a. True

b. False
