# Supplementary material for: Not a Benign (Mis)Label: Penicillin Allergy Education for the Nonallergist
Source: MedEdPORTAL. 2024 Sep 27;20:11440. doi: 10.15766/mep_2374-8265.11440 (PMC11427523; doi:10.15766/mep_2374-8265.11440)
Supplement: Supplementary file 1 — PenEd Facilitator Guide.docxPenEd Editable Survey With Answers.docxPenEd PowerPoint.pptxPenEd Student Scripts for Role-Play.docx [file mep_2374-8265.11440-s001.zip › C. PenEd PowerPoint.pptx]

## Slide 1
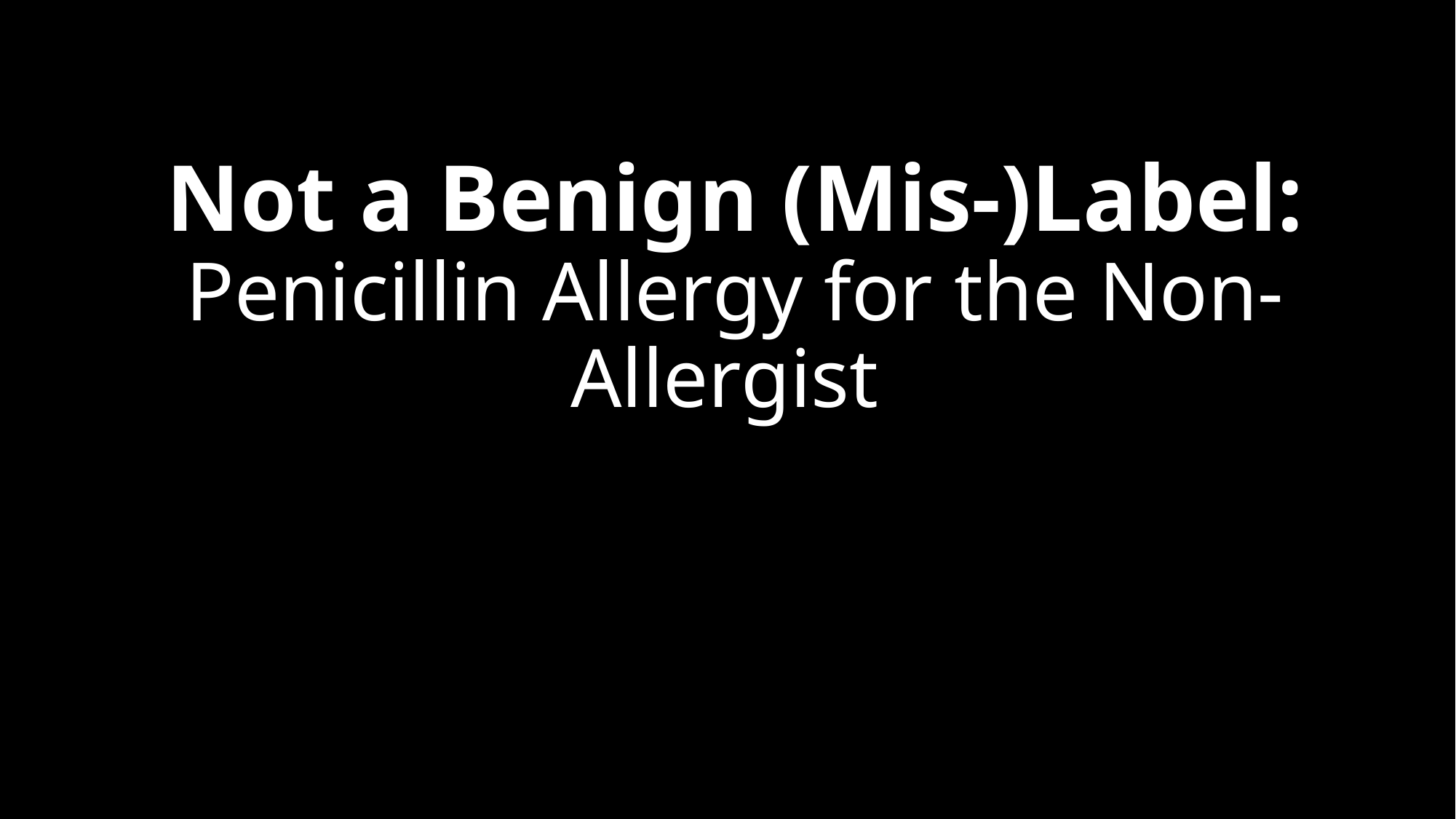

# Not a Benign (Mis-)Label: Penicillin Allergy for the Non-Allergist

## Slide 2
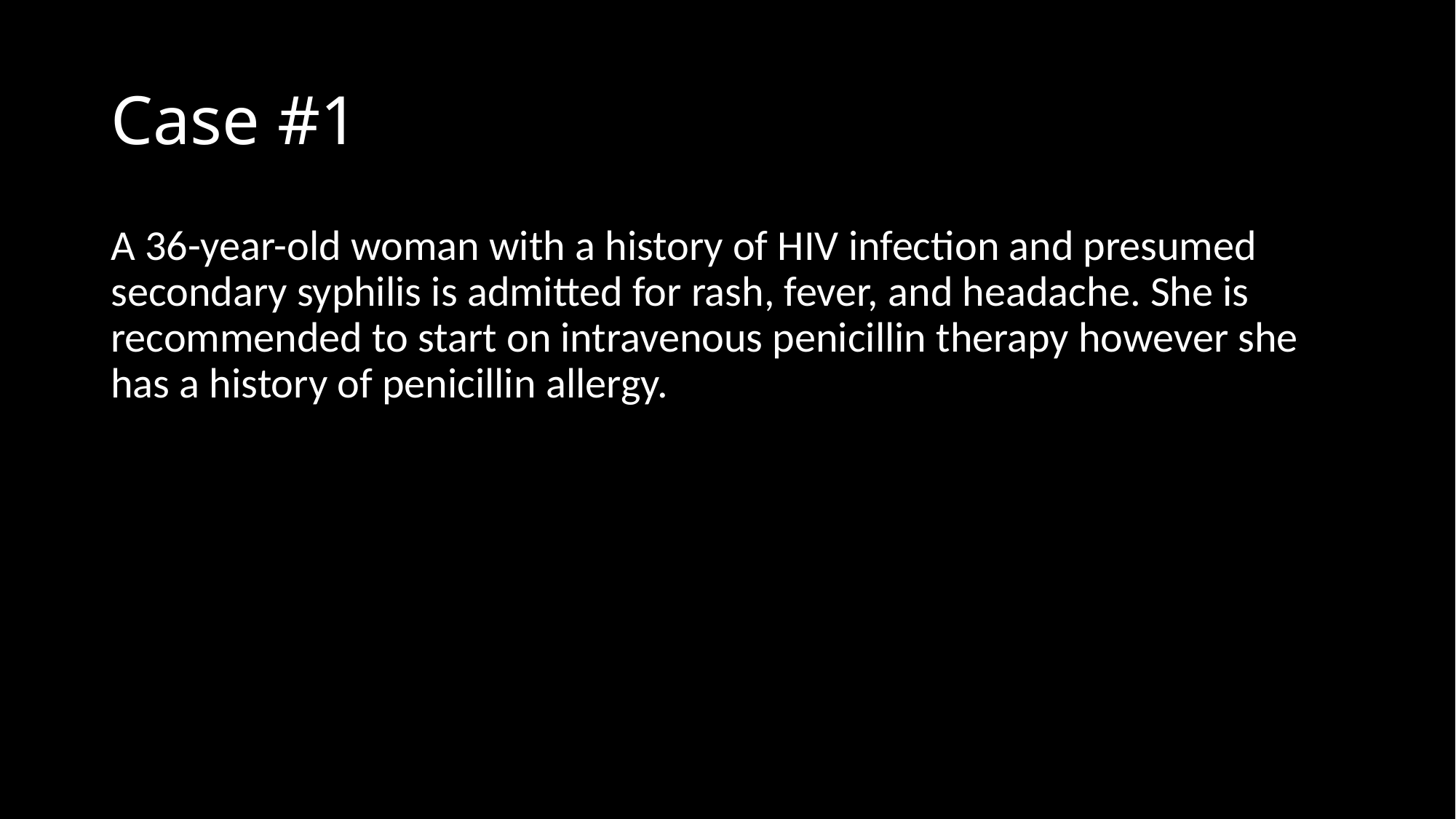

# Case #1
A 36-year-old woman with a history of HIV infection and presumed secondary syphilis is admitted for rash, fever, and headache. She is recommended to start on intravenous penicillin therapy however she has a history of penicillin allergy.

## Slide 3
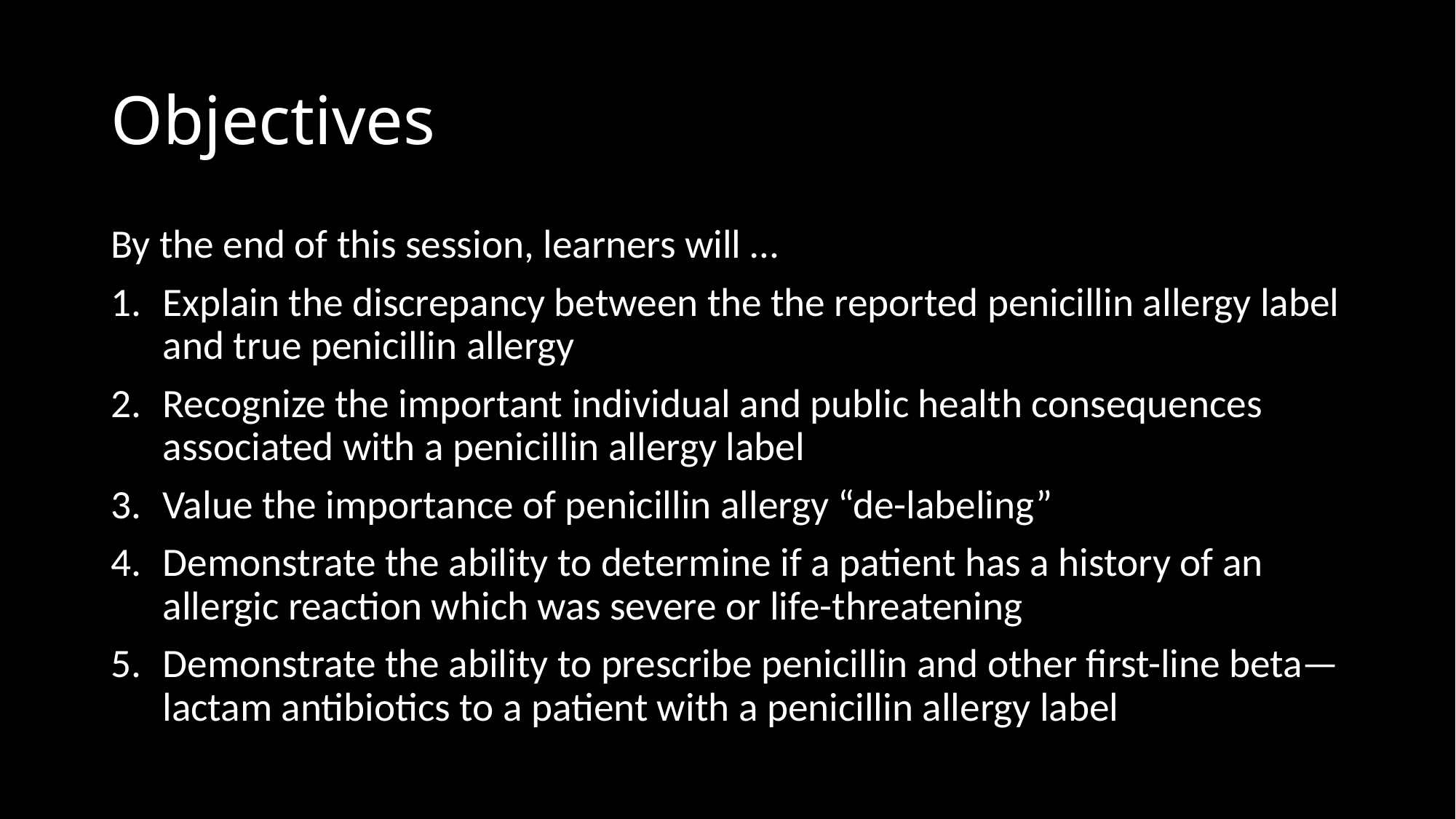

# Objectives
By the end of this session, learners will …
Explain the discrepancy between the the reported penicillin allergy label and true penicillin allergy
Recognize the important individual and public health consequences associated with a penicillin allergy label
Value the importance of penicillin allergy “de-labeling”
Demonstrate the ability to determine if a patient has a history of an allergic reaction which was severe or life-threatening
Demonstrate the ability to prescribe penicillin and other first-line beta—lactam antibiotics to a patient with a penicillin allergy label

## Slide 4
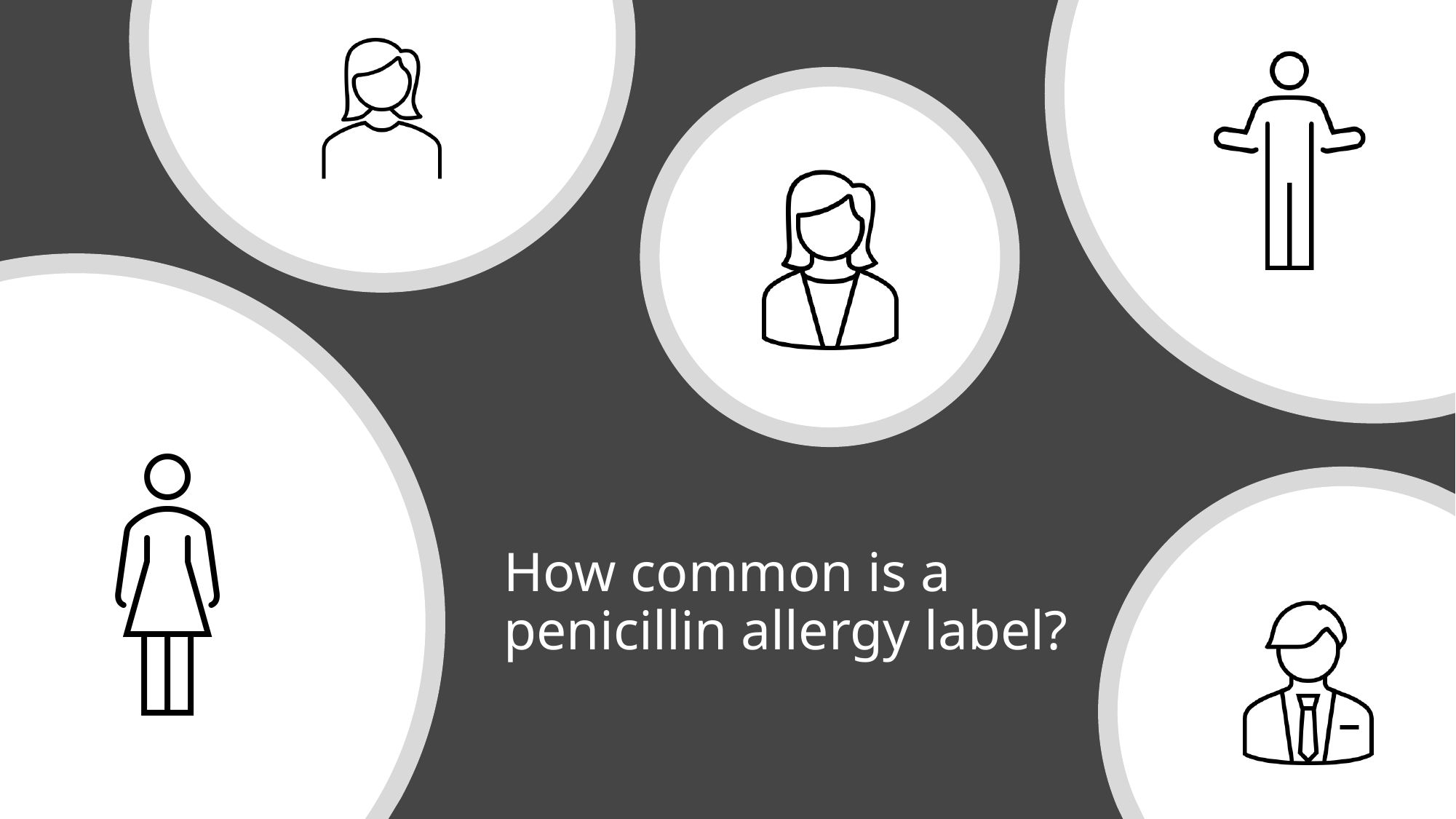

# How common is a penicillin allergy label?

## Slide 5
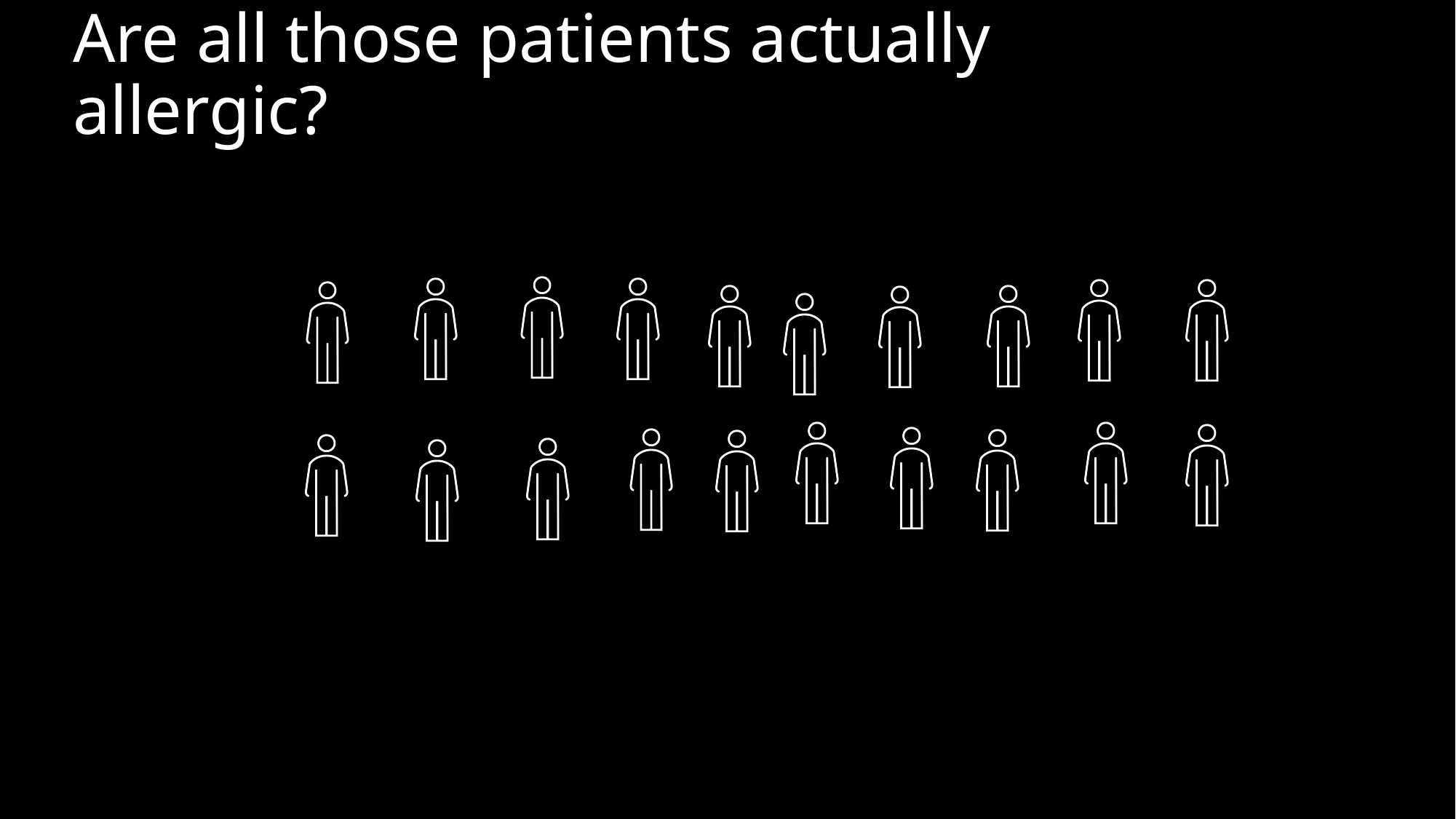

# Are all those patients actually allergic?

## Slide 6
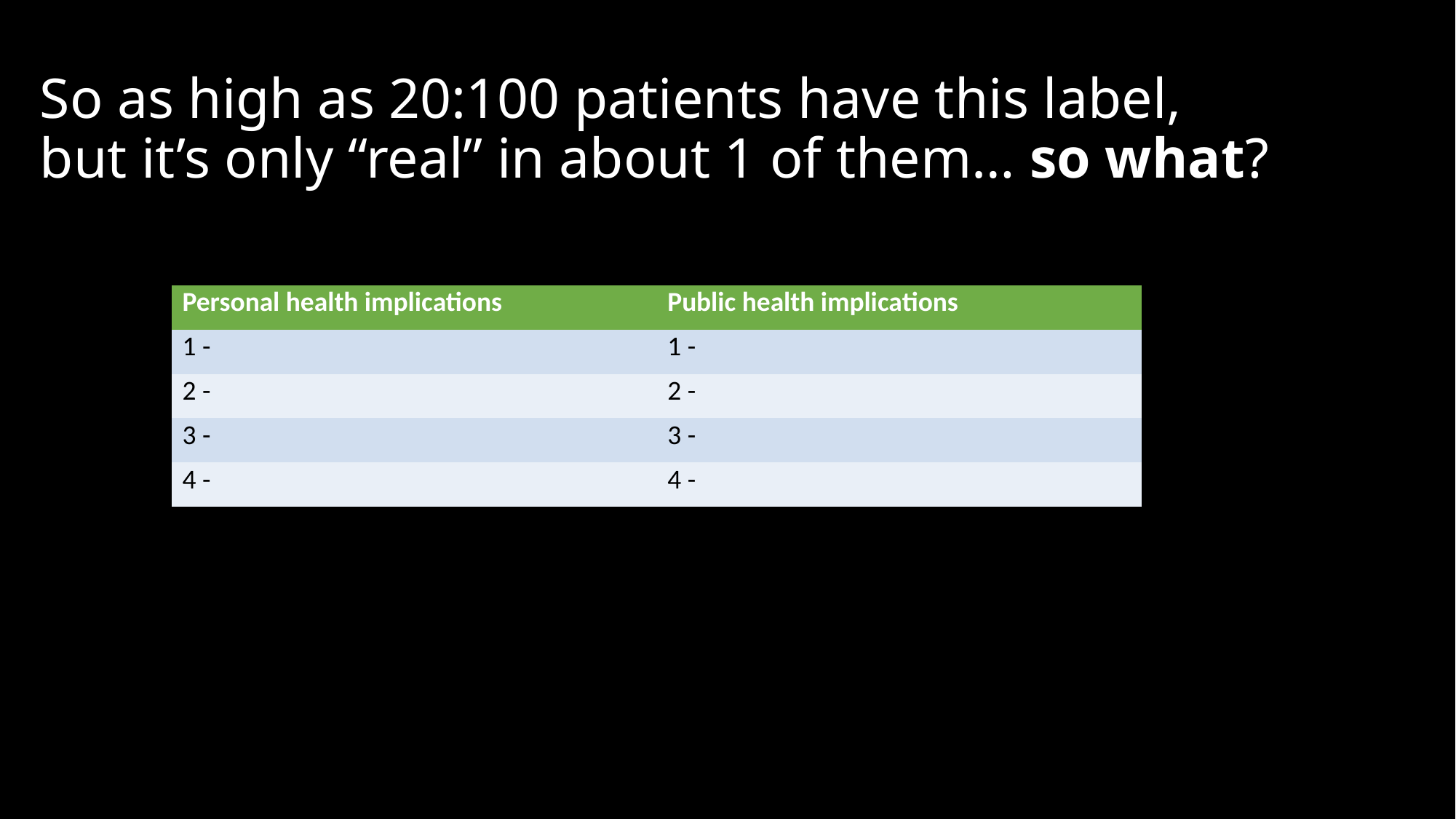

# So as high as 20:100 patients have this label, but it’s only “real” in about 1 of them… so what?
| Personal health implications | Public health implications |
| --- | --- |
| 1 - | 1 - |
| 2 - | 2 - |
| 3 - | 3 - |
| 4 - | 4 - |

## Slide 7
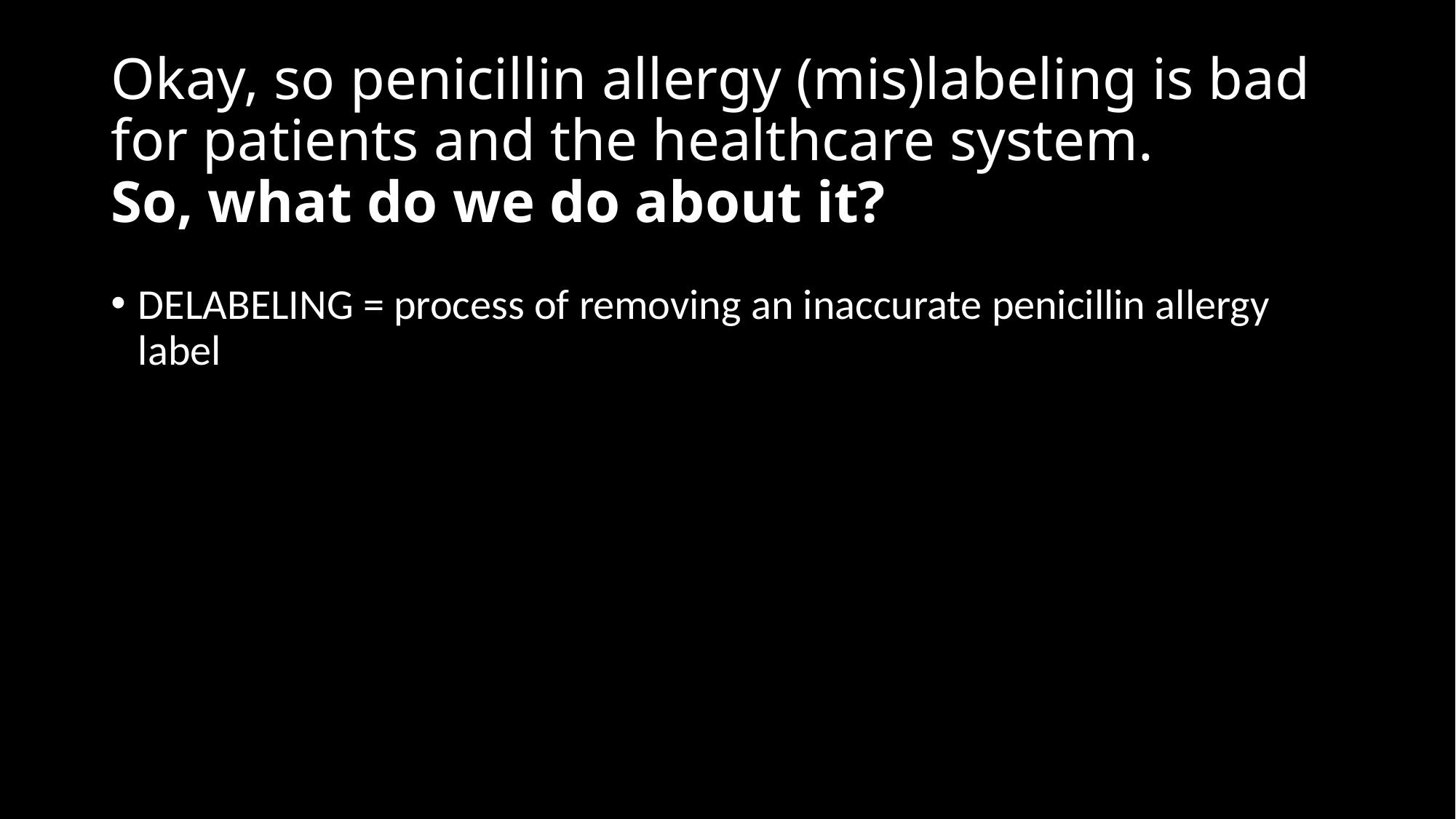

# Okay, so penicillin allergy (mis)labeling is bad for patients and the healthcare system. So, what do we do about it?
DELABELING = process of removing an inaccurate penicillin allergy label

## Slide 8
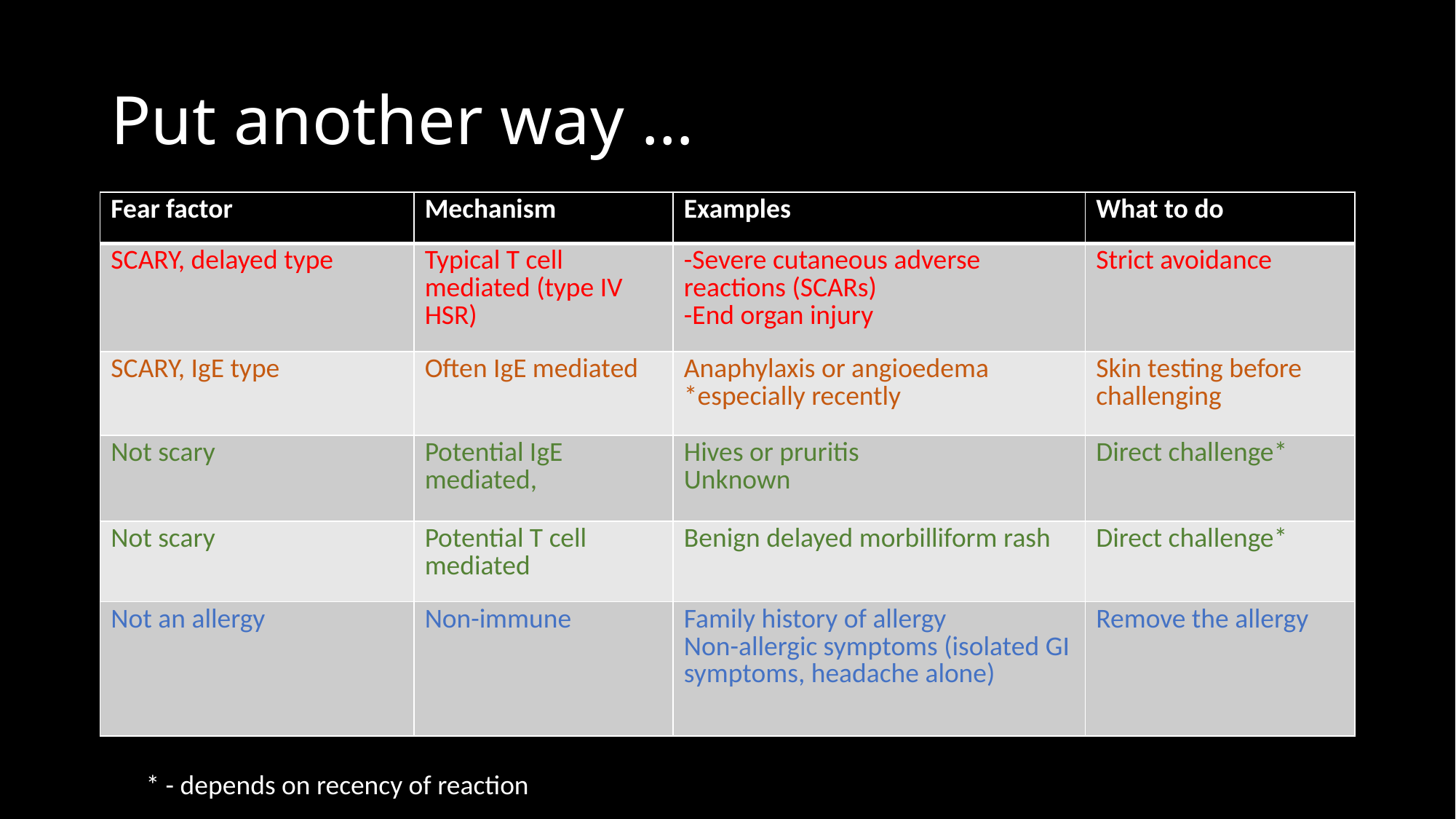

# Put another way …
| Fear factor | Mechanism | Examples | What to do |
| --- | --- | --- | --- |
| SCARY, delayed type | Typical T cell mediated (type IV HSR) | -Severe cutaneous adverse reactions (SCARs) -End organ injury | Strict avoidance |
| SCARY, IgE type | Often IgE mediated | Anaphylaxis or angioedema \*especially recently | Skin testing before challenging |
| Not scary | Potential IgE mediated, | Hives or pruritis Unknown | Direct challenge\* |
| Not scary | Potential T cell mediated | Benign delayed morbilliform rash | Direct challenge\* |
| Not an allergy | Non-immune | Family history of allergy Non-allergic symptoms (isolated GI symptoms, headache alone) | Remove the allergy |
* - depends on recency of reaction

## Slide 9
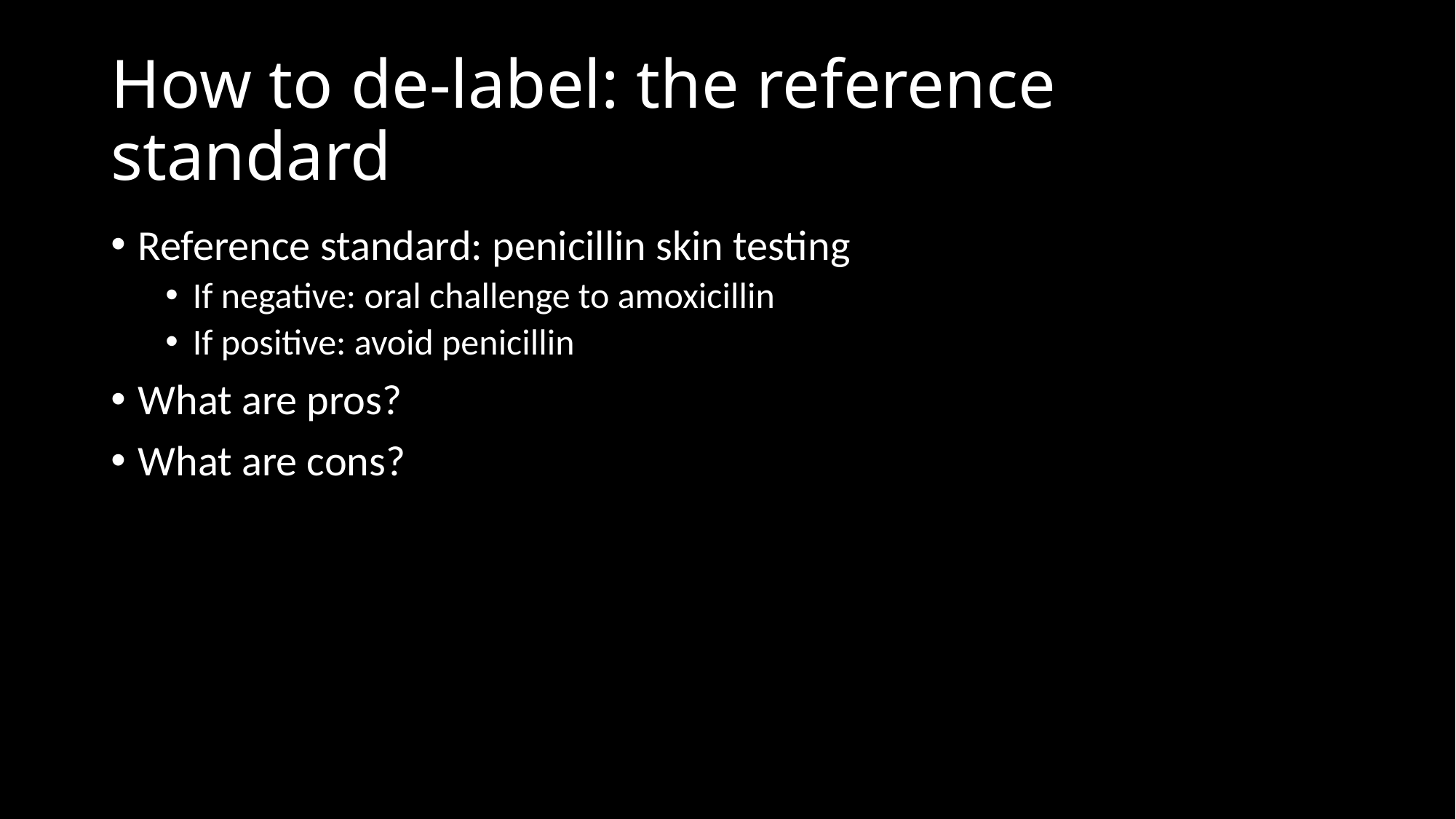

# How to de-label: the reference standard
Reference standard: penicillin skin testing
If negative: oral challenge to amoxicillin
If positive: avoid penicillin
What are pros?
What are cons?

## Slide 10
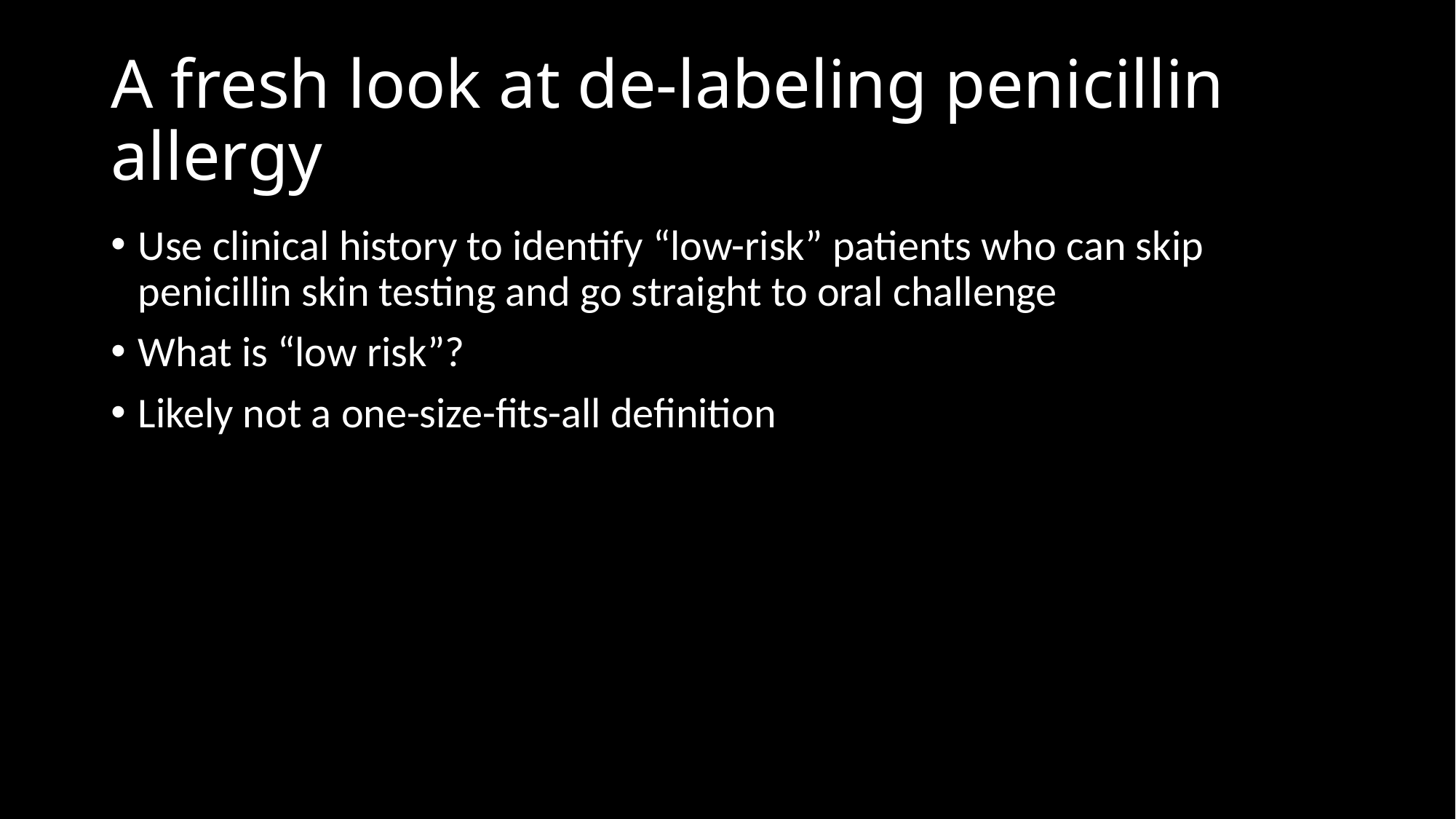

# A fresh look at de-labeling penicillin allergy
Use clinical history to identify “low-risk” patients who can skip penicillin skin testing and go straight to oral challenge
What is “low risk”?
Likely not a one-size-fits-all definition

## Slide 11
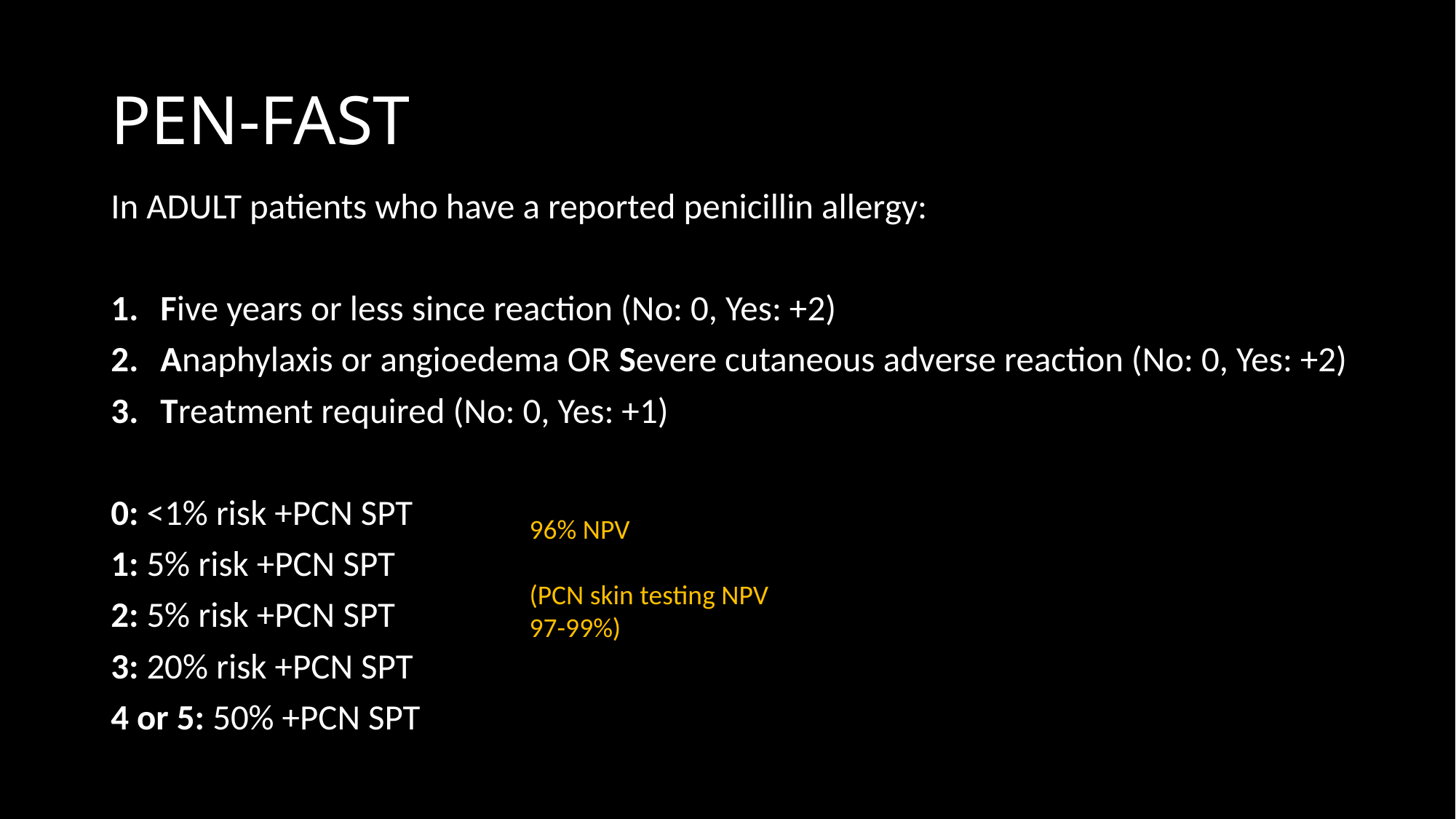

# PEN-FAST
In ADULT patients who have a reported penicillin allergy:
Five years or less since reaction (No: 0, Yes: +2)
Anaphylaxis or angioedema OR Severe cutaneous adverse reaction (No: 0, Yes: +2)
Treatment required (No: 0, Yes: +1)
0: <1% risk +PCN SPT
1: 5% risk +PCN SPT
2: 5% risk +PCN SPT
3: 20% risk +PCN SPT
4 or 5: 50% +PCN SPT
96% NPV
(PCN skin testing NPV 97-99%)

## Slide 12
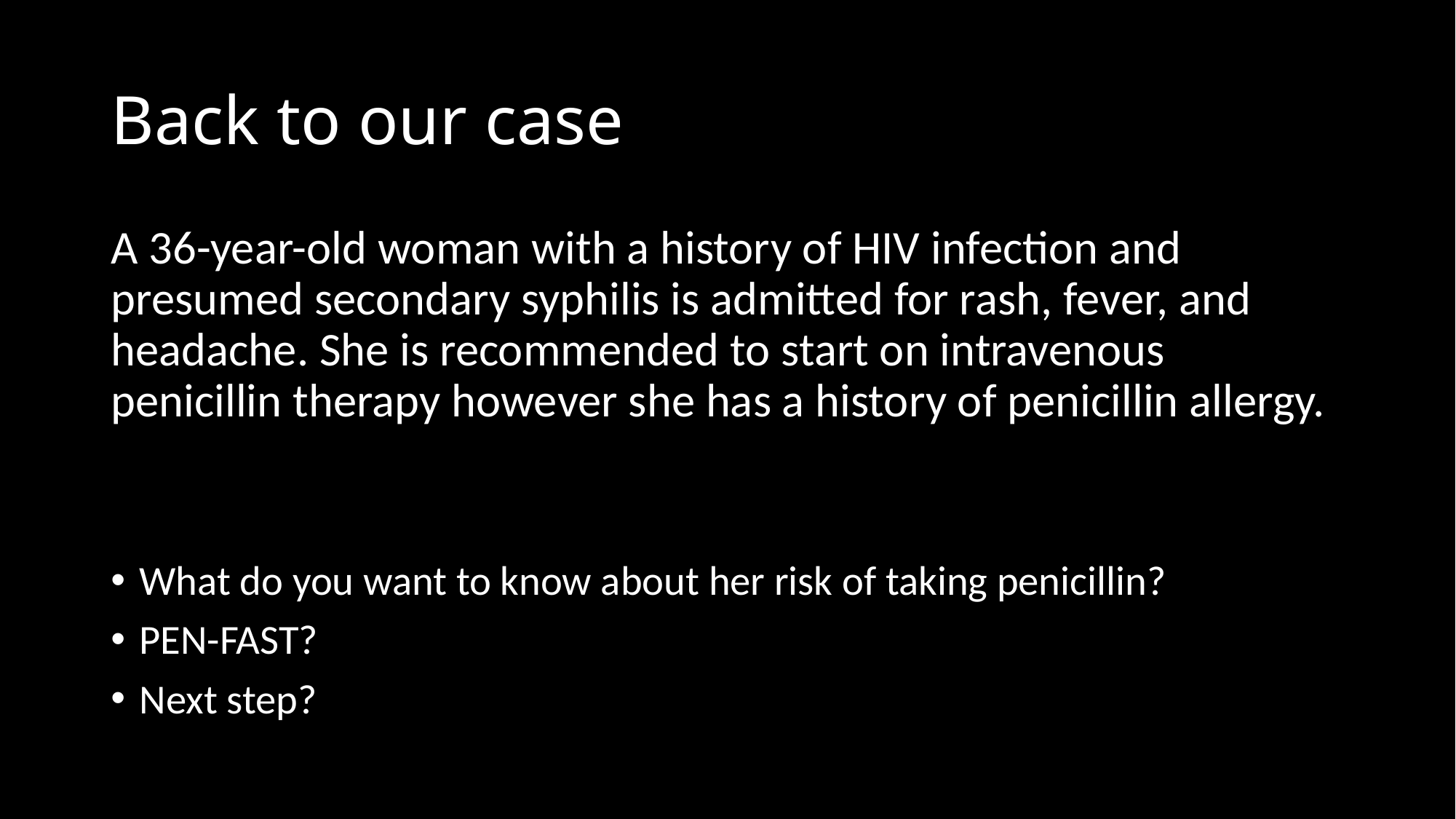

# Back to our case
A 36-year-old woman with a history of HIV infection and presumed secondary syphilis is admitted for rash, fever, and headache. She is recommended to start on intravenous penicillin therapy however she has a history of penicillin allergy.
What do you want to know about her risk of taking penicillin?
PEN-FAST?
Next step?

## Slide 13
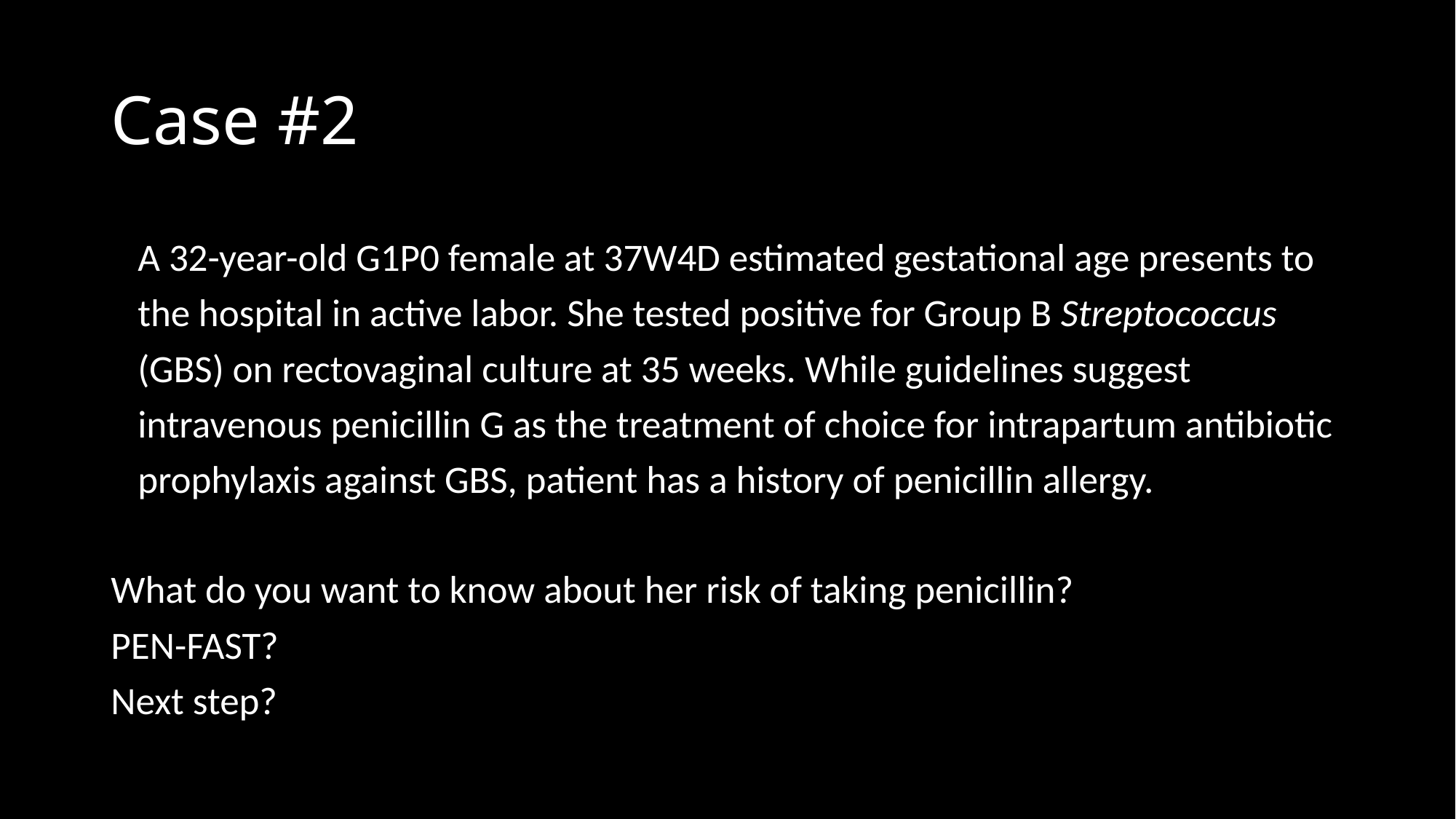

# Case #2
A 32-year-old G1P0 female at 37W4D estimated gestational age presents to the hospital in active labor. She tested positive for Group B Streptococcus (GBS) on rectovaginal culture at 35 weeks. While guidelines suggest intravenous penicillin G as the treatment of choice for intrapartum antibiotic prophylaxis against GBS, patient has a history of penicillin allergy.
What do you want to know about her risk of taking penicillin?
PEN-FAST?
Next step?

## Slide 14
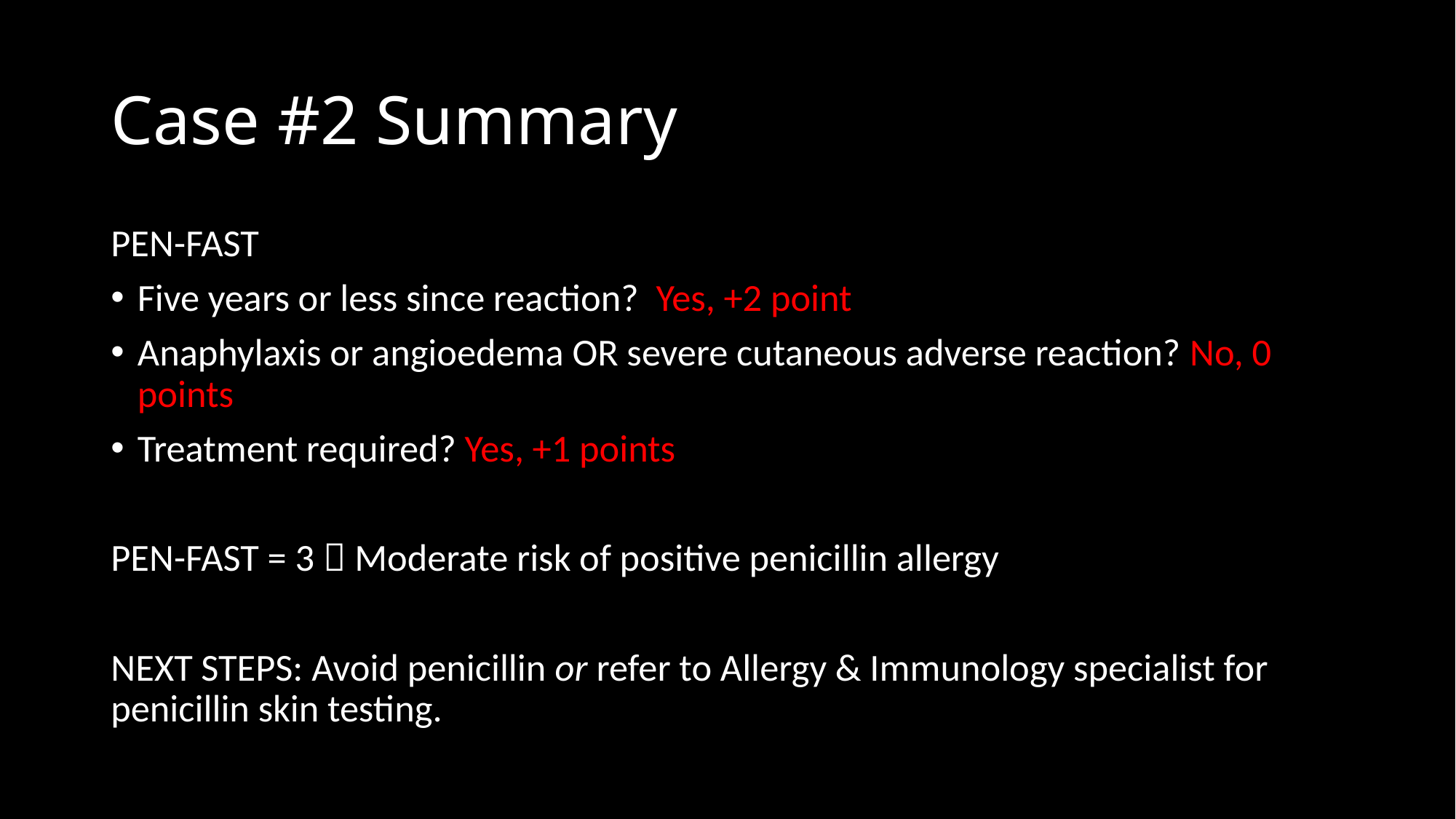

# Case #2 Summary
PEN-FAST
Five years or less since reaction? Yes, +2 point
Anaphylaxis or angioedema OR severe cutaneous adverse reaction? No, 0 points
Treatment required? Yes, +1 points
PEN-FAST = 3  Moderate risk of positive penicillin allergy
NEXT STEPS: Avoid penicillin or refer to Allergy & Immunology specialist for penicillin skin testing.

## Slide 15
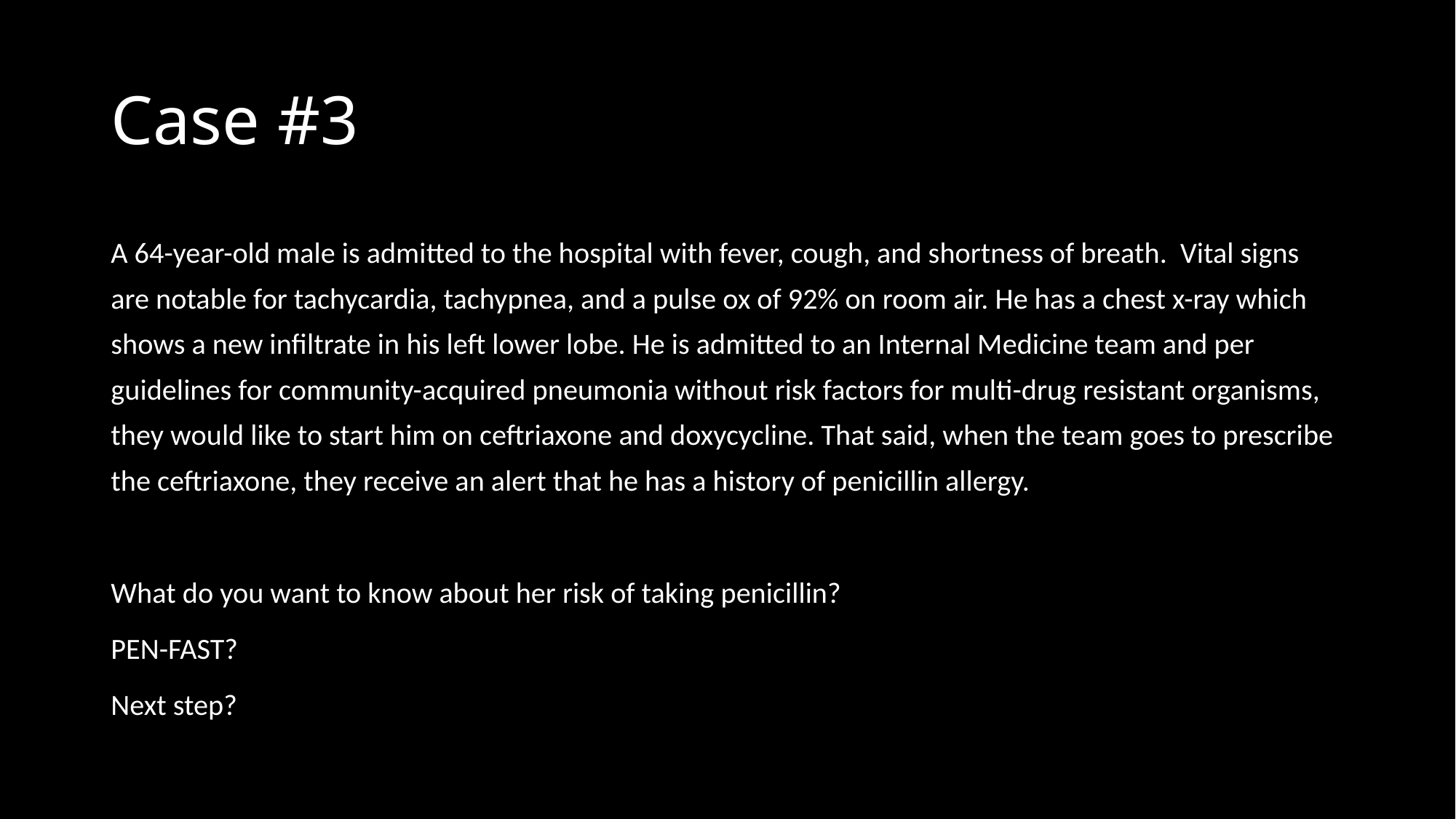

# Case #3
A 64-year-old male is admitted to the hospital with fever, cough, and shortness of breath. Vital signs are notable for tachycardia, tachypnea, and a pulse ox of 92% on room air. He has a chest x-ray which shows a new infiltrate in his left lower lobe. He is admitted to an Internal Medicine team and per guidelines for community-acquired pneumonia without risk factors for multi-drug resistant organisms, they would like to start him on ceftriaxone and doxycycline. That said, when the team goes to prescribe the ceftriaxone, they receive an alert that he has a history of penicillin allergy.
What do you want to know about her risk of taking penicillin?
PEN-FAST?
Next step?

## Slide 16
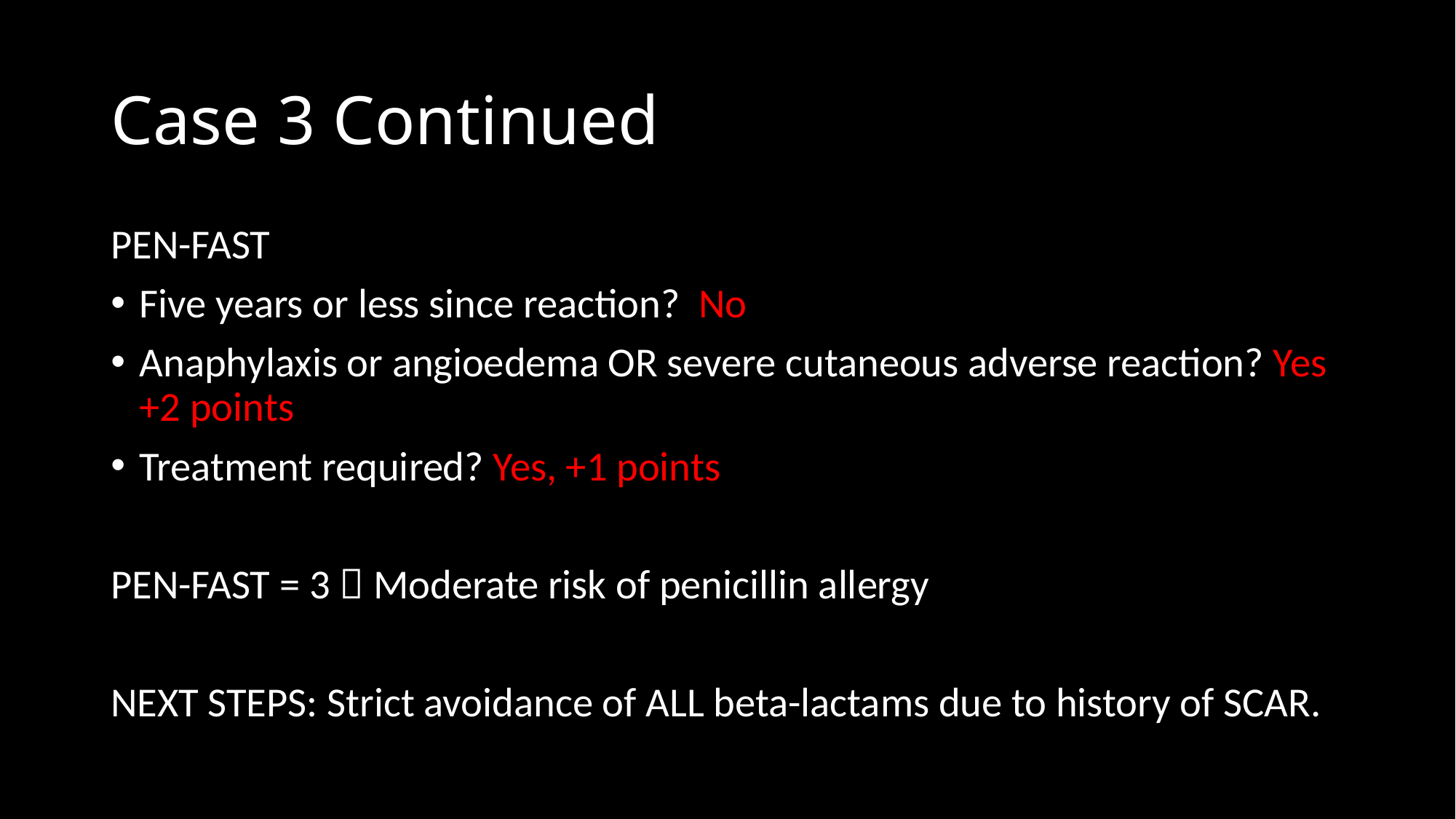

# Case 3 Continued
PEN-FAST
Five years or less since reaction? No
Anaphylaxis or angioedema OR severe cutaneous adverse reaction? Yes +2 points
Treatment required? Yes, +1 points
PEN-FAST = 3  Moderate risk of penicillin allergy
NEXT STEPS: Strict avoidance of ALL beta-lactams due to history of SCAR.

## Slide 17
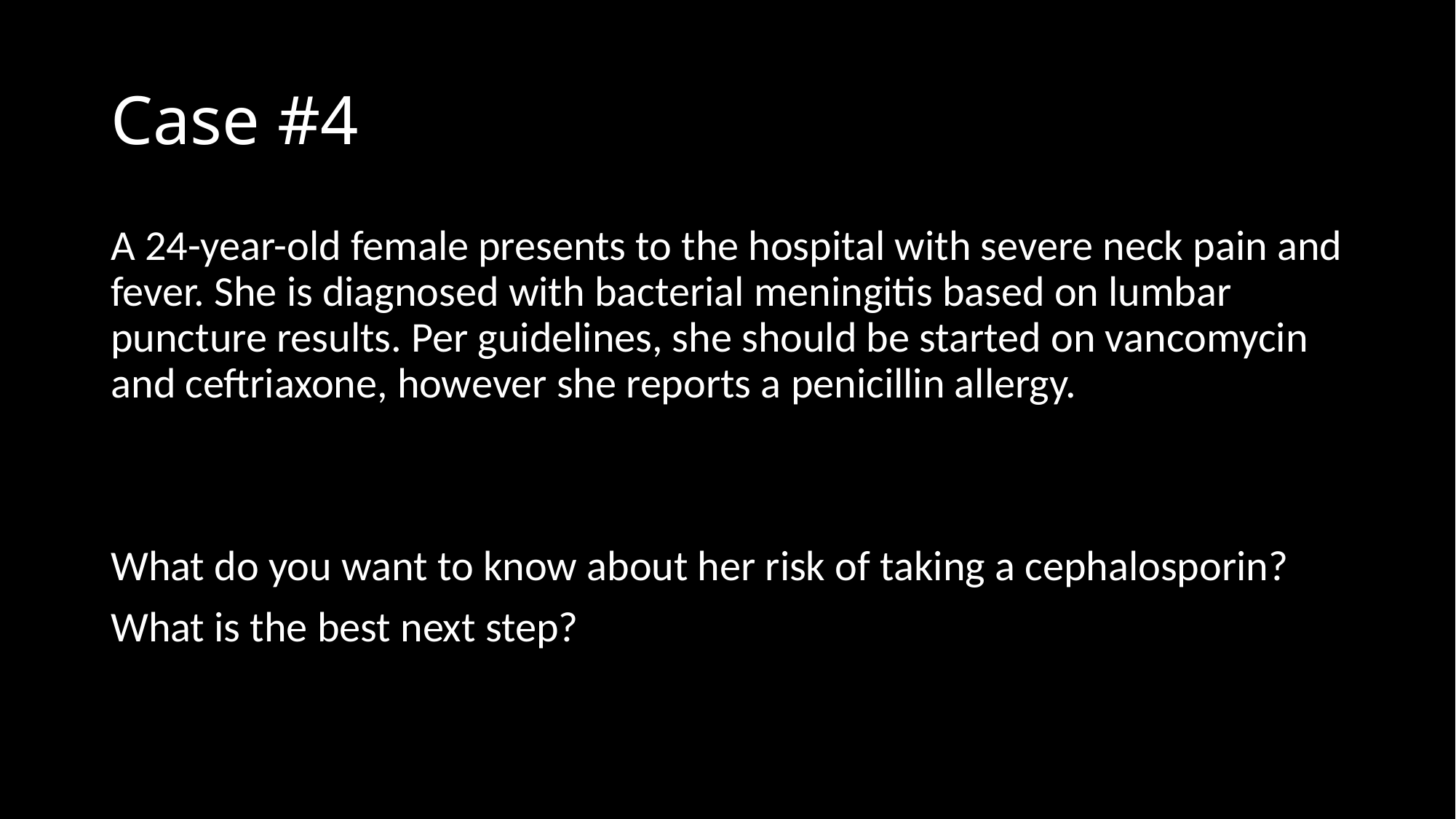

# Case #4
A 24-year-old female presents to the hospital with severe neck pain and fever. She is diagnosed with bacterial meningitis based on lumbar puncture results. Per guidelines, she should be started on vancomycin and ceftriaxone, however she reports a penicillin allergy.
What do you want to know about her risk of taking a cephalosporin?
What is the best next step?

## Slide 18
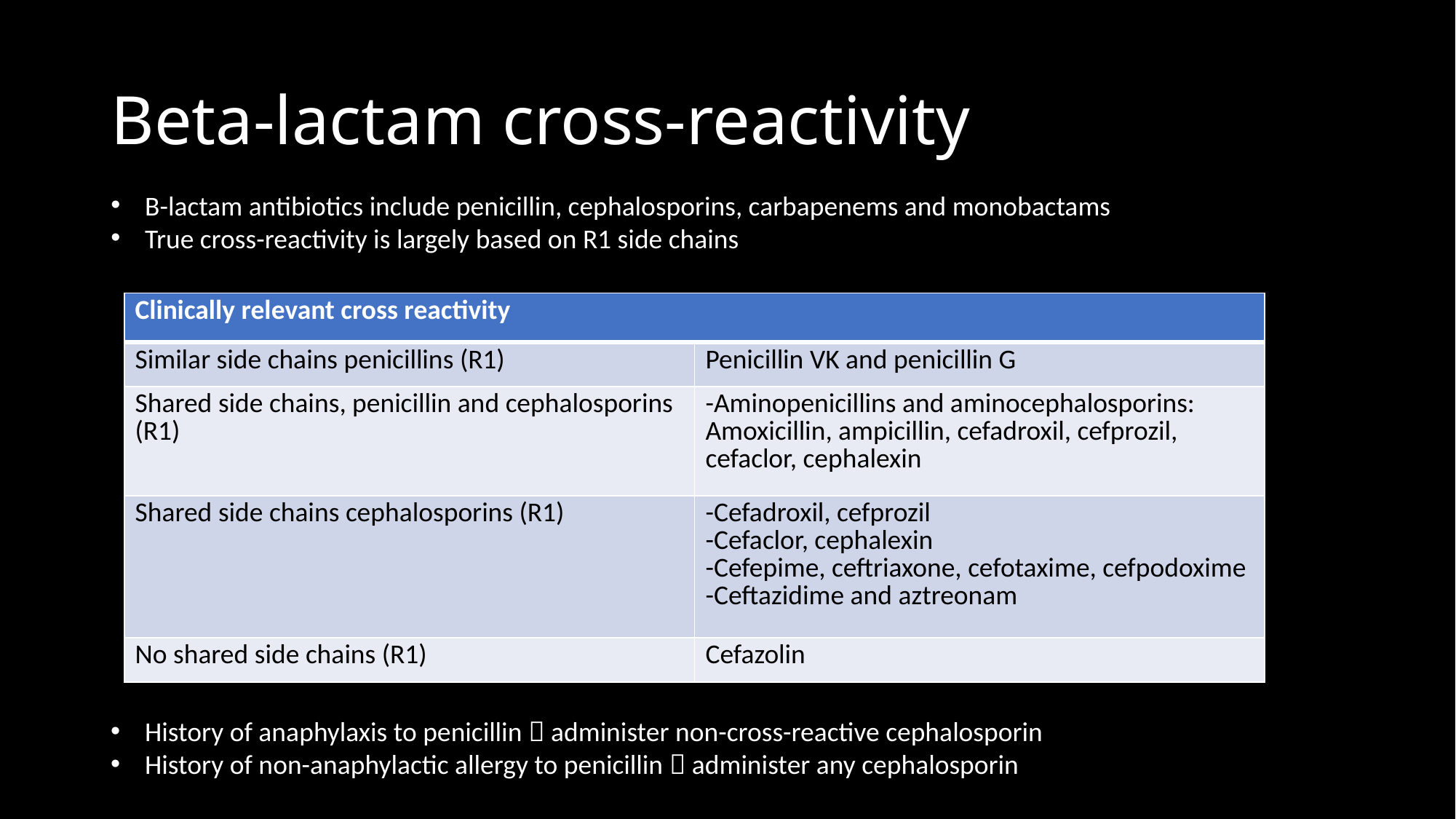

# Beta-lactam cross-reactivity
B-lactam antibiotics include penicillin, cephalosporins, carbapenems and monobactams
True cross-reactivity is largely based on R1 side chains
| Clinically relevant cross reactivity | |
| --- | --- |
| Similar side chains penicillins (R1) | Penicillin VK and penicillin G |
| Shared side chains, penicillin and cephalosporins (R1) | -Aminopenicillins and aminocephalosporins: Amoxicillin, ampicillin, cefadroxil, cefprozil, cefaclor, cephalexin |
| Shared side chains cephalosporins (R1) | -Cefadroxil, cefprozil -Cefaclor, cephalexin -Cefepime, ceftriaxone, cefotaxime, cefpodoxime -Ceftazidime and aztreonam |
| No shared side chains (R1) | Cefazolin |
History of anaphylaxis to penicillin  administer non-cross-reactive cephalosporin
History of non-anaphylactic allergy to penicillin  administer any cephalosporin

## Slide 19
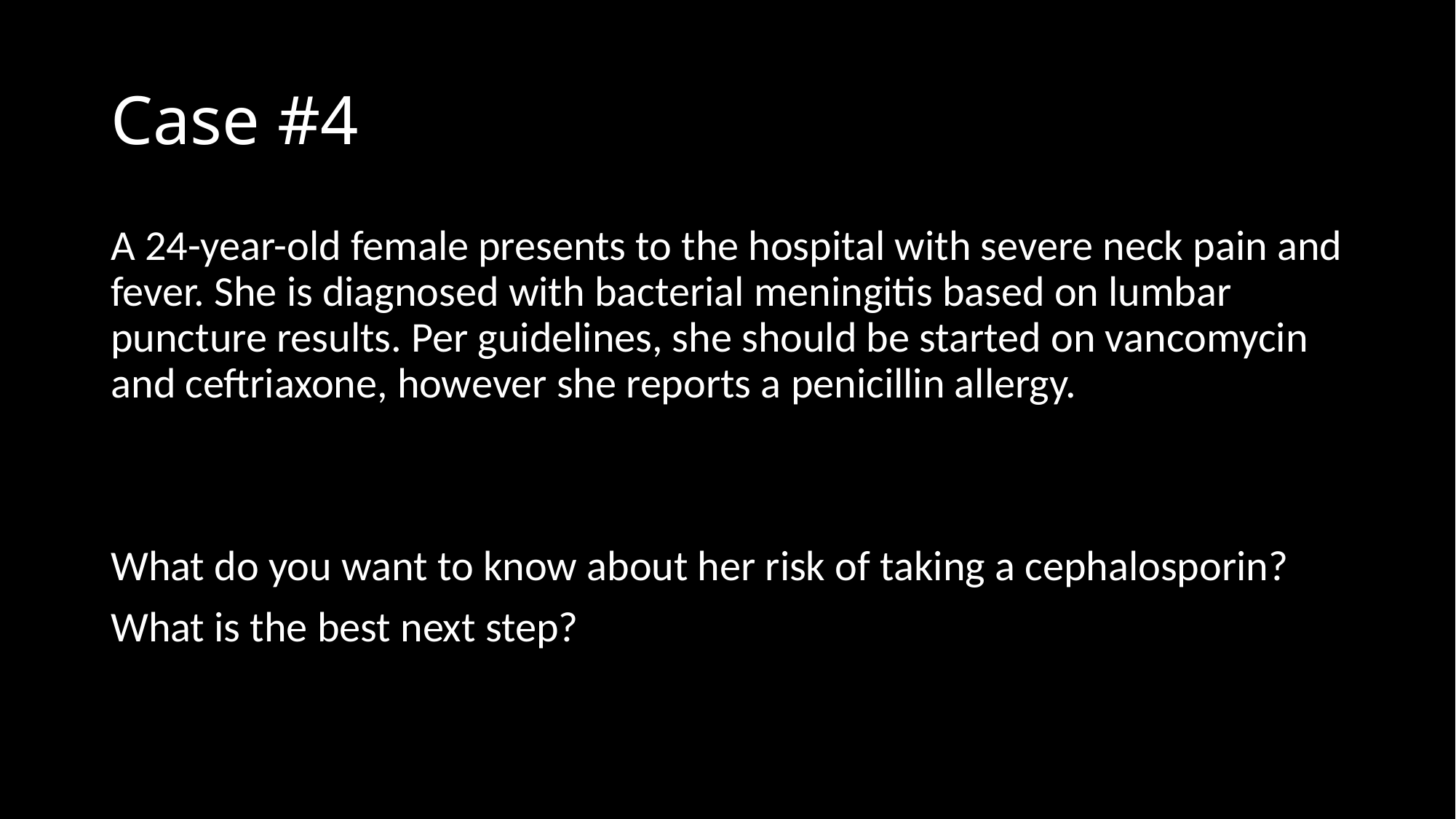

# Case #4
A 24-year-old female presents to the hospital with severe neck pain and fever. She is diagnosed with bacterial meningitis based on lumbar puncture results. Per guidelines, she should be started on vancomycin and ceftriaxone, however she reports a penicillin allergy.
What do you want to know about her risk of taking a cephalosporin?
What is the best next step?

## Slide 20
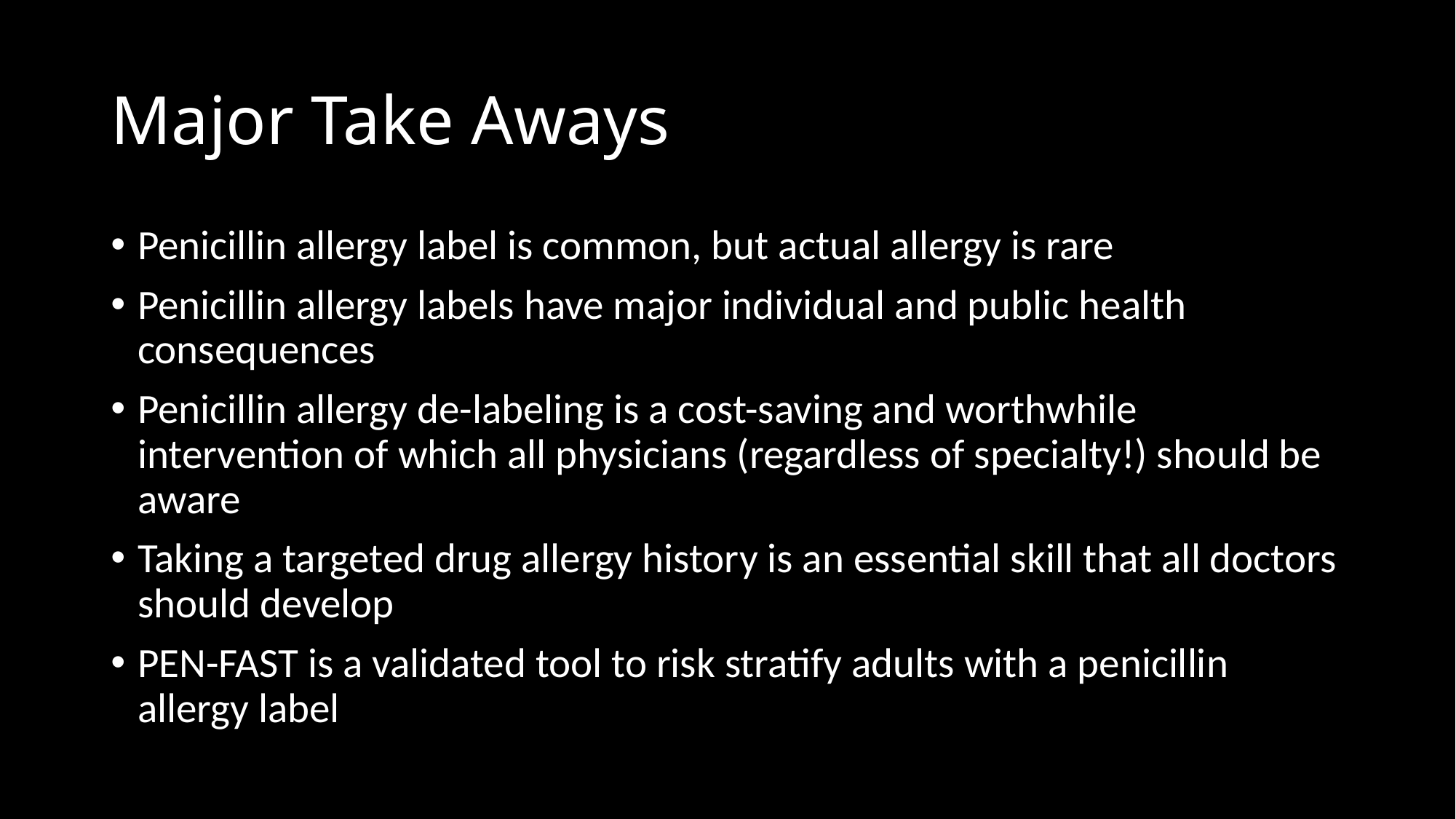

# Major Take Aways
Penicillin allergy label is common, but actual allergy is rare
Penicillin allergy labels have major individual and public health consequences
Penicillin allergy de-labeling is a cost-saving and worthwhile intervention of which all physicians (regardless of specialty!) should be aware
Taking a targeted drug allergy history is an essential skill that all doctors should develop
PEN-FAST is a validated tool to risk stratify adults with a penicillin allergy label
