# Supplementary material for: Not a Benign (Mis)Label: Penicillin Allergy Education for the Nonallergist
Source: MedEdPORTAL. 2024 Sep 27;20:11440. doi: 10.15766/mep_2374-8265.11440 (PMC11427523; doi:10.15766/mep_2374-8265.11440)
Supplement: Supplementary file 1 — PenEd Facilitator Guide.docxPenEd Editable Survey With Answers.docxPenEd PowerPoint.pptxPenEd Student Scripts for Role-Play.docx [file mep_2374-8265.11440-s001.zip › A. PenEd Facilitator Guide.docx]

**PenEd Facilitator Guide**

**Introduction**

The Penicillin Allergy for the Non-Allergist (PenEd) curriculum is aimed to improve learners’ self-efficacy and knowledge regarding adult penicillin allergy. This workshop begins with a short didactic section, and then pivots to active learning techniques including case-based learning and role playing.

**Facilitator Preparation**

Familiarize with the materials including the PowerPoint, student cases, and surveys. The student cases and/or surveys may be printed out.

**Learning Objectives:**

By the end of this session, learners will:

1. Explain the discrepancy between the reported penicillin allergy label and true penicillin allergy.
2. Recognize the important individual and public health consequences associated with a penicillin allergy label.
3. Value the importance of the practice of penicillin allergy “de-labeling.”
4. Demonstrate the ability to determine if a patient has a history of an allergic reaction which was severe or life-threatening.
5. Demonstrate the ability to prescribe penicillin and other beta-lactam antibiotics to patients with a penicillin allergy label.

**Curriculum Schedule (Approximate)**

0-5 minutes (5 minutes total): Introduction, Optional pre-survey

5-30 minutes (25 minutes total): Didactic Presentation (Slides 1-11). Approximate slide times below (Can adapt accordingly)

- Slide 2: 1 minute
- Slide 3: 1 minute
- Slide 4: 2 minutes
- Slide 5: 2 minutes
- Slide 6: 5 minutes
- Slide 7: 2 minutes
- Slide 8: 4 minutes
- Slide 9: 2 minutes
- Slide 10: 3 minutes
- Slide 11: 3 minutes

30-35 minutes (7 minutes total): Case #1 (Slide 12)

32-39 minutes (7 minutes total): Case #2 (Slide 13)

39-45 minutes (7 minutes total): Case #3 (Slide 14)

45-52 minutes (7 minutes total): Case #4 (Slide 15-17)

52-60 minutes (8 minutes total): Wrap up, Optional post-survey (Slide 18)

**Facilitator Guide:**

This workshop includes 2 components: didactics followed by case-based learning and role-playing.

The didactic portion begins with case #1, which is intended to pique students’ interest. After reviewing the objectives of the workshop, the facilitator discusses the epidemiology and health consequences of a penicillin allergy label. The facilitator then reviews drug allergy de-labeling, which historically has relied on skin testing, but more recently has used history-based risk stratification. After reviewing a basic approach to drug allergy classification, the facilitator should introduce PEN-FAST, which is a validated decision tool to risk stratify penicillin allergy in adults. Medical students should be encouraged to access this tool on their smartphones via MDCALC (if available). The facilitator should then pivot back to case #1, and role-play with students to familiarize them with the PEN-FAST clinical decision tool.

The facilitator should then break students up into groups of 2, and pass out case #2 and case #3. For case #2, one student should pretend to be the patient (and thus have access to the distributed patient history) and one student should pretend to be the physician (or allergist).. Then, for case #3, the students should switch roles while staying in the same pairs. Each case, as indicated above, takes approximately 5-7 minutes for students to role play and use the PEN-FAST tool to risk stratify the history (low risk, moderate risk, high risk). While the Student Role Play Guides (Appendix C) are sparse and only contain enough information to answer the PEN-FAST tool, they can easily be elaborated if desired by a future facilitator.

After both case #2 and case #3, the entire class should come back together and share how they used the PEN-FAST tool to risk stratify the clinical history. Because case #4 covers new information regarding beta-lactam prescribing for patients with penicillin allergy labels, this case should be worked together as an entire class, again with the facilitator pretending to be the patient.

Finally, the facilitator should review the major takeaways and, if applicable, fill out the post-survey. Depending on time and preference, the facilitator can choose to review the answers to the post-survey.
