# Supplementary material for: Not a Benign (Mis)Label: Penicillin Allergy Education for the Nonallergist
Source: MedEdPORTAL. 2024 Sep 27;20:11440. doi: 10.15766/mep_2374-8265.11440 (PMC11427523; doi:10.15766/mep_2374-8265.11440)
Supplement: Supplementary file 1 — PenEd Facilitator Guide.docxPenEd Editable Survey With Answers.docxPenEd PowerPoint.pptxPenEd Student Scripts for Role-Play.docx [file mep_2374-8265.11440-s001.zip › D. PenEd Student Scripts for Role-Play.docx]

**Case 2: Patient Script**

You will pair up with another student who will play the physician interviewing the patient to determine the PEN-FAST score. You will be playing the role of the patient using the following script:

WHEN was the allergic reaction? Response: “4 years ago.”

In response to the physician’s direct questions, you will answer “yes” or “no” to the following reactions and/or symptoms of these reactions:

- Hives? YES, 4 years ago
- Blistering? NO
- Mucosal involvement? NO
- Angioedema? NO
- Anaphylaxis? NO
- Require treatment? YES

**Case 2: Provider Script**

You will pair up with another student who will play the patient. Your job is to play the physician and determine the PEN-FAST score. You will be playing the role of the patient using the following script:

- When was the allergic reaction?
- Did you have hives?
- Did you have blistering?
- Did you have mucosal involvement?
- Did you have angioedema?
- Did you have anaphylaxis?
- Did you require treatment?

**Case 3 Patient Script:**

You will pair up with another student who will play the physician interviewing the patient to determine the PEN-FAST score. You will be playing the role of the patient using the following script:

WHEN was the allergic reaction? Response: “50 years ago or more”

In response to the physician’s direct questions, you will answer “yes” or “no” to the following reactions and/or symptoms of these reactions:

- Hives? NO
- Blistering? YES. Took long-term penicillin for rheumatic fever however developed blistering rash which required hospitalization
- Mucosal involvement? Yes
- Angioedema? NO
- Anaphylaxis? NO
- Require treatment? YES

**Case 3: Provider Script**

You will pair up with another student who will play the patient. Your job is to play the physician and determine the PEN-FAST score. You will be playing the role of the patient using the following script:

- When was the allergic reaction?
- Did you have hives?
- Did you have blistering?
- Did you have mucosal involvement?
- Did you have angioedema?
- Did you have anaphylaxis?
- Did you require treatment?
