# Supplementary material for: Not a Benign (Mis)Label: Penicillin Allergy Education for the Nonallergist
Source: MedEdPORTAL. 2024 Sep 27;20:11440. doi: 10.15766/mep_2374-8265.11440 (PMC11427523; doi:10.15766/mep_2374-8265.11440)
Supplement: Supplementary file 1 — PenEd Facilitator Guide.docxPenEd Editable Survey With Answers.docxPenEd PowerPoint.pptxPenEd Student Scripts for Role-Play.docx [file mep_2374-8265.11440-s001.zip › _Educational Summary Report_11440.pdf]

MedEdPORTAL® The AAMC Journal of Teaching and Learning Resources

Original Publication Open Access

Not a Benign (Mis)Label: Penicillin Allergy Education for the Nonallergist

Jessica Plager, MD*, William B. Cutrer, MEd, MD

*Corresponding author: jessicaplager@gmail.com

Abstract

Introduction: Up to 20% of the US population carries a penicillin allergy label; however, over 95% of those patients can safely tolerate penicillin. This discrepancy has important personal and public health consequences. There is no published curriculum for medical trainees that covers penicillin allergy history taking, risk assessment, and antibiotic prescribing. **Methods:** We created a 60-minute, interactive curriculum that targeted medical students during their internal medicine rotation. We employed learning strategies including didactics, case-based learning, and role-playing. We compared self-efficacy and knowledge before and after the intervention using paired *t* tests. **Results:** A total of 28 medical students participated, with 25 completing both the pre- and postworkshop surveys. There was a statistically significant improvement in student-rated preparedness to prescribe antibiotics to patients with a penicillin allergy label ($p < .001$) and determine whether a patient has a history of an allergic reaction that was severe or life-threatening ($p < .001$). There was additionally a statistically significant increase in students' perception that penicillin allergy labels carry important health consequences ($p = .005$), as well as increase in their total knowledge scores ($p = .006$). **Discussion:** The workshop employs adult learning techniques to improve self-efficacy and knowledge regarding penicillin allergy in medical students. Further work is needed to refine the curriculum, seek external validity, and determine the impact of this workshop on clinical outcomes.

Keywords
Penicillin, Delabeling, Case-Based Learning, Allergy & Immunology

Educational Objectives

By the end of this session, learners will:

1. Explain the discrepancy between the reported penicillin allergy label and true penicillin allergy.
2. Recognize the important individual and public health consequences associated with a penicillin allergy label.
3. Value the importance of the practice of penicillin allergy delabeling.
4. Demonstrate the ability to determine if a patient has a history of an allergic reaction that was severe or life-threatening.
5. Demonstrate the ability to prescribe penicillin and other beta-lactam antibiotics to patients with a penicillin allergy label.

Citation:
Plager J, Cutrer WB. Not a benign (mis)label: penicillin allergy education for the nonallergist. *MedEdPORTAL*. 2024;20:11440.
https://doi.org/10.15766/mep_2374-8265.11440

Introduction

At first glance, an allergy to penicillin may seem common, even innocuous. However, it is neither. While up to 20% of the population engaged in medical care reports such an allergy, less than 5% of those labeled are truly allergic.[1] This penicillin allergy (mis)label is not benign; when clinicians eschew beta-lactam antibiotics in favor of other antibiotics, they often deviate from standard of care antibiotics, thereby risking inferior infection outcomes, antibiotic-related complications, and antimicrobial resistance.[2-5] As just one example, a retrospective cohort study of over 8,000 postsurgical patients (hip arthroplasty, knee arthroplasty, hysterectomy, colon surgery and coronary bypass grafting), revealed that patients with a penicillin allergy label received less cefazolin and more clindamycin, vancomycin, and gentamicin compared with those who did not report a penicillin allergy label; this inferior antibiotic choice conferred a 50% higher risk of surgical site infection.[5] Recognizing the negative personal and public health outcomes associated with a penicillin allergy label, the 2022 Drug Allergy Practice Parameters recommend proactive penicillin allergy delabeling, which refers to the process of removing an inaccurate penicillin allergy label. Furthermore, the authors advocate that "strong efforts should be made

Copyright © 2024 Plager and Cutrer This is an open-access publication distributed under the terms of the Creative Commons Attribution license.

to educate about the benefits of delabeling to patients and clinicians."[6]

Joining this effort, this article shares a dedicated penicillin allergy curriculum that is designed for medical students yet adaptable to many other learners. While allergists are typically seen as drug allergy content experts and have access to specialized penicillin allergy skin testing, the majority of penicillin allergy labels could be safely and accurately evaluated by nonallergists utilizing evidence-based risk-stratification tools. With this curriculum, future and current clinicians can learn how to risk-stratify a penicillin allergy history, thereby gaining the knowledge and skills to either delabel the penicillin allergy or safely prescribe other beta-lactam antibiotics.

PenEd, a term specific to this curriculum, is an abbreviation for penicillin education, and rifts off PEN-FAST, which is a penicillin allergy risk-stratification tool utilized in the curriculum and further discussed in the educational summary report. The PenEd curriculum was devised utilizing Kern's six-step model for curricular development.[7] To our knowledge, this is the first penicillin allergy curriculum for medical trainees, including medical students.

Methods

Devising the Curriculum

Although most health practitioners take a drug allergy history, and although up to one in five patients engaged in medical care report a penicillin allergy, there is surprisingly little published about drug allergy education in general, and penicillin allergy in particular.[1] Following Kern's six-step model for curricular development,[7] we performed a global needs assessment through performing a literature search on the state of drug allergy medical education. Using PubMed, ERIC, Embase, and Web of Science, we identified a mere six papers that focused on drug allergy medical education. While the majority (four of six) concentrated on drug allergy emergencies (e.g., anaphylaxis, angioedema, and infusion syndromes), one used an educational program in conjunction with an antibiotic prescribing guideline to evaluate providers' antibiotic prescribing knowledge, and one discussed penicillin allergy assessment and skin testing as it pertained to pharmacy students.[7-13] Based on our review of the literature, there is no currently published curriculum on penicillin allergy that exists for medical students, residents, and/or fellows.

To identify the specific knowledge gaps of the medical students at Vanderbilt University School of Medicine (VUSM), we collaborated with the Vanderbilt Educational Design and Informatics Team to perform a targeted needs assessment (step 2 of Kern's model). Specifically, we searched the VUSM digital curriculum map utilizing the Medical Subject Headings (MeSH) "drug-related side effects and adverse reactions" and "drug hypersensitivity." We identified 85 teaching sessions whose learning objectives matched onto these MeSH terms, and, after eliminating those sessions focusing solely on nonimmune mediated side effects, we identified only one session that covered drug allergy. That said, this session discussed drug allergy only within the context of the Gell-Coombs framework and did not specifically cover penicillin allergy or how to risk-stratify a drug allergy history.

We sought to incorporate both cognitive and affective objectives into our PenEd curriculum (step 3 of Kern's model). From a knowledge perspective, we hoped to have students recognize the discrepancy between real and reported penicillin allergy and appreciate the negative health consequences associated with a penicillin allergy label. On an attitudinal level, we hoped students would rank as important the practice of penicillin allergy delabeling. Finally, we aimed to train students to use the validated PEN-FAST clinical decision tool to determine if a patient has a severe penicillin allergy history, and to use beta-lactam cross-reactivity tables to safely prescribe antibiotics to a patient with a penicillin allergy label.[14] These objectives mapped onto the VUSM competencies, including, but not limited to, Medical Knowledge 3 (demonstrate knowledge of the sciences that support other specialty fields as they relate to one's own practice), Patient Care 1 (perform a problem-focused or complete history) and Patient Care 6 (utilize informatics and health information technology in support of patient care).[15]

Setting and Participants

PenEd targeted VUSM second-year medical students during their internal medicine core clerkship rotation. The educational session was held during the noon conference hour, during which time the medical students were released from service obligations. There was no required prework prior to the session. The Vanderbilt University Medical Center Institutional Review Board deemed the educational session exempt from review.

This session was held twice for a total of 28 medical students (roughly 25% of the medical school class). This medical education project was completed in the primary author's second and final year of allergy and immunology fellowship as her research project. After spending the first part of her second year planning the project, she spent the second part of the year implementing the curriculum and analyzing the results. Because of this time constraint, only 25% of the medical school class was ultimately able to participate in the curriculum. The primary author, who has

Copyright © 2024 Plager and Cutrer This is an open-access publication distributed under the terms of the Creative Commons Attribution license.

since graduated from the VUMC fellowship, intends to deliver the curriculum to learners at other institutions.

Instructional Strategy and Implementation
When selecting our educational strategies (step 4 of Kern's model), we aimed to create a curriculum that not only flowed from our educational objectives, but also was aligned with adult learning theory. As detailed in the PenEd Facilitator Guide (Appendix A), the first 5 minutes of the lesson was dedicated to filling out the presurvey (Appendix B). Following students' completion of the presurvey, there was a 15-minute didactic session that was administered largely via PowerPoint (Appendix C). The didactic portion started with a clinical case designed to emphasize the topic's clinical relevance, and then went on to discuss the prevalence and health implications of a penicillin allergy label. After reviewing ways to categorize a drug allergy history, students learned how to use PEN-FAST, which is a validated online decision tool used to risk-stratify adults with a penicillin allergy history. Using the PEN-FAST tool, students reexamined case 1 and practiced risk-stratifying a drug allergy history in a group. The students then broke into pairs and alternated role-playing (physician and patient) with case 2 and case 3, utilizing student scripts for role-play (Appendix D), so that each student could practice individually taking a drug allergy history and utilizing the PEN-FAST tool. After reviewing case 2 and 3 together as a group and clarifying any questions, the facilitator reviewed case 4, which examined new information about how to prescribe cephalosporins in patients with penicillin allergy labels. Finally, during the last 5-10 minutes of the session, students complete the postsurvey (Appendix B). We did not review answers of the pre- or postsurvey with students due to time constraints.

Facilitators
One second-year allergy and immunology (A/I) fellow with training in medical education delivered the didactic lecture and facilitated the case-based learning sessions for both PenEd sessions. As previously mentioned, we developed a PowerPoint, facilitator guide, as well as student handouts for cases 2 and 3. The session was designed such that future instructors would not need to attend a training session prior to delivering the workshop and could simply review the facilitator guide.

Assessment
We developed a survey using the REDCap survey tool to assess the effectiveness of the PenEd workshop in achieving the objectives. While no validated survey instrument on penicillin allergy existed, we utilized three questions from previously studied instruments.[9] Because those prior questions were written in a true/false format, we kept the same format for the remainder of the questions. The survey was developed by A/I physicians, and piloted with VUSM medical students on their internal medicine clerkship. The survey was administered at the start of the PenEd workshop (pretest) and following the conclusion of the final case (posttest). While the survey was not anonymous, students were informed that it would in no way impact their grade, and that the facilitator would be blinded to the results. The survey included a visual analog scale for questions 2-4, which covered students' perception of their preparedness as well as attitude regarding penicillin allergy. It also included a true/false section (questions 5-13), which assessed knowledge of drug allergy, with specific attention to penicillin allergy. While quality and effectiveness were not included as part of the survey questions, the facilitator indirectly measured these via student participation and engagement as well as self-rated preparedness survey questions, respectively.

Data Analysis
We utilized SAS version 9 (SAS Institute) for data analysis. REDCap assigned a numerical value to the visual analog scale questions, and we compared pretest and pos-test scores using a dependent t test. We then calculated students' total number correct (0-9) on the true/false questions and used a dependent t test to compare pretest and posttest mean scores.

Results

Of the 28 medical students who completed the PenEd training, 25 (89%) completed both the pre- and postworkshop surveys. The results from the pretest ($M = 32.9$, $SD = 25.0$) and posttest ($M = 80.3$, $SD = 12.3$) indicated that medical students increased, on average, 51.2 points in their self-rated preparedness to prescribe antibiotics for patients with a penicillin allergy, $t(24) = 9.99$, $p < .001$ (Figure 1). Furthermore, results from the pretest ($M = 50.0$, $SD = 27.0$) and posttest ($M = 89.9$, SD = 10.3) showed that medical students increased, on average, 42.3 points on their self-rated preparedness to determine if a patient had a history of an allergic reaction that was severe or life-threatening $t(24) = 8.78$, $p < .001$ (Figure 2). Finally, results from the pretest ($M = 71.0$, $SD = 25.1$) and posttest ($M = 91.6$, $SD = 9.5$) indicated that students increased, on average, 21.2 points in their belief that having a penicillin allergy has important personal health and public health consequences $t(24) = 3.99$, $p = .005$ (Figure 3). In comparing the pretest ($M = 7.5$, $SD = 1.1$) and posttest ($M = 8.5$, $SD = 0.8$) knowledge scores, results indicate that students improved an average of 1.0 points on a 0-9 scale $t(24) = 3.98$, $p = .006$. For students' results on individual questions, see the Table.

Copyright © 2024 Plager and Cutrer This is an open-access publication distributed under the terms of the Creative Commons Attribution license.

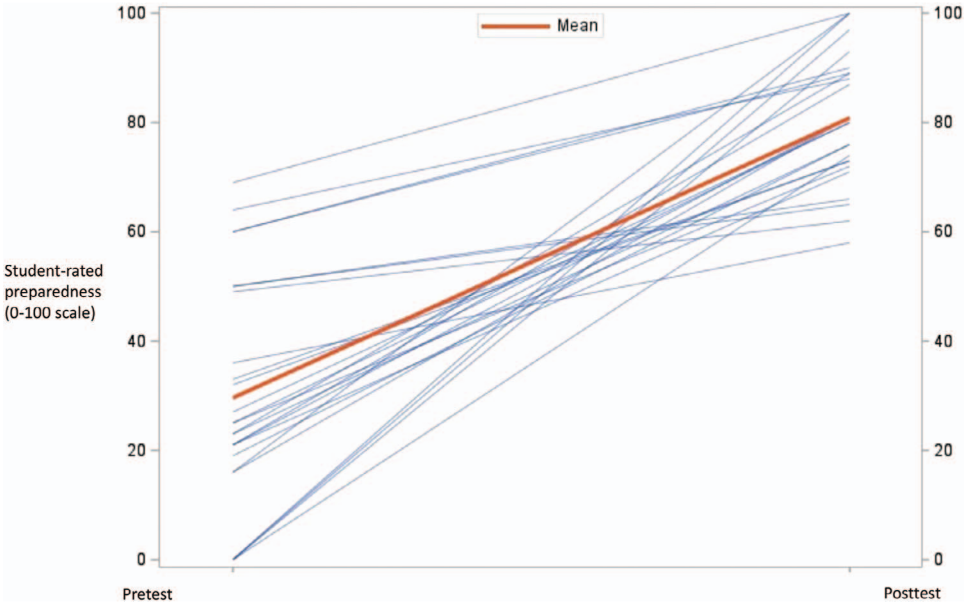

Figure 1. Student-rated preparedness to prescribe antibiotics to patients with penicillin allergy. The results from the pretest (M = 32.9, SD = 25.0) and posttest (M = 80.3, SD = 12.3) indicated that medical students increased, on average, 51.2 points in their self-rated preparedness to prescribe antibiotics for patients with a penicillin allergy, $t(24) = 9.99$, $p < .001$.

Discussion

Students who participated in the PenED curriculum experienced statistically significant improvement in self-efficacy and knowledge about penicillin allergy. By incorporating various learning strategies including case-based learning, role-playing, and didactics, PenEd utilized adult learning techniques to teach medical students about penicillin allergy. This session was delivered by a single facilitator over the course of 1 hour and did not require learner self-directed preparation.

While the PenEd workshop was delivered to medical students during their internal medicine clerkship, it could easily be delivered to any health professional that prescribes antibiotics

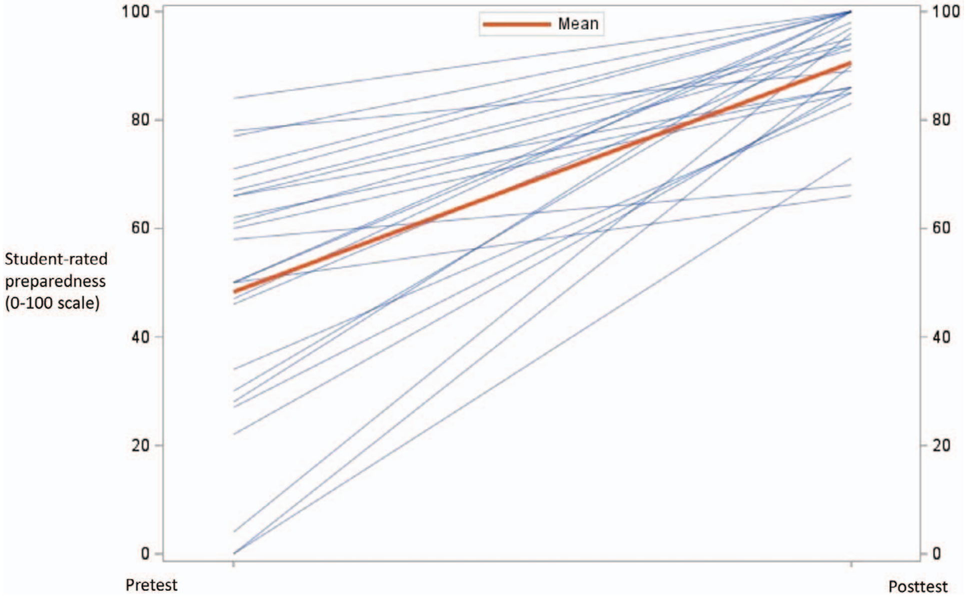

Figure 2. Student-rated preparedness to determine if patient has a history of an allergic reaction that is severe or life-threatening. Results from the pretest (M = 50.0, SD = 27.0) and posttest (M = 89.9, SD = 10.3) showed that medical students increased, on average, 42.3 points on their self-rated preparedness to determine if a patient had a history of an allergic reaction that was severe or life-threatening, $t(24) = 8.78$, $p < .001$.

Copyright © 2024 Plager and Cutrer This is an open-access publication distributed under the terms of the Creative Commons Attribution license.

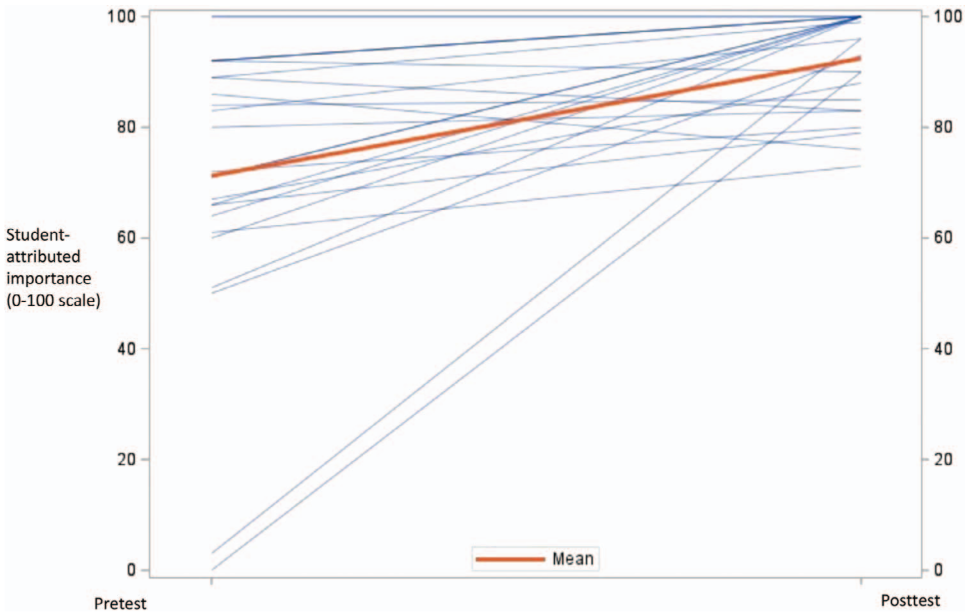

Figure 3. Student perception that penicillin allergy label has important personal and public health consequences. Results from the pretest (M = 71.0, SD = 25.1) and posttest (M = 91.6, SD = 9.5) indicate that students increased, on average, 21.2 points in their belief that having a penicillin allergy has important personal health and public health consequences, $t(24) = 3.99$, $p = .005$.

to adult patients. Because the management of penicillin allergy differs between children and adults (e.g., PEN-FAST is validated only for adults), it would need to be adapted to cover pediatric drug allergy.

Table. Student Pre- and Posttest Knowledge Scores

Survey Question	Pretest Performance (%)	Posttest Performance (%)
Penicillin allergy is not forever.	96%	96%
Skin testing is a valid tool for assessing penicillin allergy.	96%	96%
Over 90% of individuals with a penicillin allergy label can safely take penicillin.	96%	96%
A family history of drug allergy is not a risk factor for penicillin allergy.	57%	100%
Patients with penicillin reaction histories such as headache or isolated gastrointestinal symptoms do not require further testing before prescribing penicillin.	64%	100%
Patients with a history of penicillin allergy should be referred for drug allergy evaluation prior to needing antibiotics	85%	93%
Delabeling is cost-saving.	96%	96%
Skin testing should be used for patients with a penicillin allergy history of anaphylaxis or recent reaction that is suspected to be IgE-mediated.	71%	85%
SCAR reaction to beta-lactam is an absolute contraindication to receiving future beta-lactams	89%	85%
Total score[a]	84%	94%

Abbreviations: IgE, Immunoglobulin E; SCAR, severe cutaneous adverse reaction.
[a]Mean percentage of correct answers.

Despite being constructed as a 60-minute session with no prework, PenEd demonstrated that students were able to achieve the desired cognitive and affective objectives (outlined in methods section). Students not only improved in their overall test scores and self-rated efficacy (cognitive domain), but also grew to appreciate the myriad negative health outcomes associated with a penicillin allergy label (affective domain). While our postsurvey did not directly assess effectiveness, we believe that by significantly increasing students' perceived preparedness to risk-stratify a drug allergy history and prescribe antibiotics to patients with a penicillin allergy, our curriculum is indeed quite effective. As a single, 60-minute session, this workshop could easily be integrated into a clinical rotation (as was the case in our study) or held during a noon conference or orientation. Additional time could be added to the session to allow for review of posttest questions.

We acknowledge potential limitations to this workshop. It was implemented within a single medical school with only 25 participants, which could limit the generalizability of our findings. From an operational standpoint, not all medical schools may have an allergy physician to facilitate this session. We developed surveys using content experts and piloted it with VUSM second-year medical students, but did not perform additional validity or reliability testing, nor were questions reviewed by a person with survey writing expertise. Because we wanted to utilize previously studied questions that were

Copyright © 2024 Plager and Cutrer This is an open-access publication distributed under the terms of the Creative Commons Attribution license.

already in a true/false format, we standardized our entire survey to also be in this format. For future implementation, we may want to adapt the survey to be in multiple-choice questions, which are not prone to the same disadvantages of true/false questions (harder to write, 50% chance of correct answer, etc.). In that light, the survey would indubitably benefit from review by someone with survey writing expertise. Moreover, while we assessed curriculum quality via student participation, future postsurveys could incorporate questions regarding curriculum quality. Further work is also needed to demonstrate retention of knowledge and feelings of self-efficacy.

At present, we can see some future changes that would benefit the curriculum itself. For instance, one could experiment with elaborating the student scripts for role-play (Appendix D) in order to incorporate more information than that which is simply required to answer the PEN-FAST decision tool. While students did improvise other details of the case during real life implementation of the curriculum, this could be more standardized in the future.

It is important to acknowledge that while students improved in their knowledge and preparedness regarding penicillin allergy, it is unclear how this will translate to clinical outcomes. Future directions could include studying clinical outcome measures including delabeling, use of beta-lactams, and referral to allergy/immunology specialists.

In summary, PenEd is a highly effective single-session workshop that incorporates adult learning techniques to teach learners about penicillin allergy in adults. The curriculum could be easily adapted to different learners and programs.

Appendices

A. PenEd Facilitator Guide.docx

B. PenEd Editable Survey With Answers.docx

C. PenEd PowerPoint.pptx

D. PenEd Student Scripts for Role-Play.docx

All appendices are peer reviewed as integral parts of the Original Publication.

Jessica Plager, MD: Clinical Fellow, Division of Allergy, Pulmonology, and Critical Care, Vanderbilt University Medical Center; ORCID: https://orcid.org/0009-0003-3272-8768

William B. Cutrer, MEd, MD: Associate Professor of Pediatrics and Anesthesiology, Vanderbilt University Medical Center; ORCID: https://orcid.org/0000-0003-1538-9779

Acknowledgments
We would like to acknowledge Nanette Dendy, MD, the internal medicine clerkship director, as well as Kirsty Clark, PhD, who helped with our statistics.

Disclosures
None to report.

Funding/Support
None to report.

Ethical Approval
The Vanderbilt University Medical Center Institutional Review Board deemed further review of this project not necessary.

References

1. Castells M, Khan DA, Phillips EJ. Penicillin allergy. *N Engl J Med*. 2019;381(24):2338-2351. https://doi.org/10.1056/NEJMra1807761
2. Kaminsky LW, Ghahramani A, Hussein R, Al-Shaikhly T. Penicillin allergy label is associated with worse clinical outcomes in bacterial pneumonia. *J Allergy Clin Immunol Pract*. 2022;10(12): 3262-3269. https://doi.org/10.1016/j.jaip.2022.08.027
3. Mattingly TJ II, Fulton A, Lumish RA, et al. The cost of self-reported penicillin allergy: a systematic review. *J Allergy Clin Immunol Pract*. 2018;6(5):1649-1654.e4. https://doi.org/10.1016/j.jaip.2017.12.033
4. Blumenthal KG, Lu N, Zhang Y, Li Y, Walensky RP, Choi HK. Risk of meticillin resistant *Staphylococcus aureus* and *Clostridium difficile* in patients with a documented penicillin allergy: population based matched cohort study. *BMJ*. 2018;361:k2400. https://doi.org/10.1136/bmj.k2400
5. Blumenthal KG, Ryan EE, Li Y, Lee H, Kuhlen JL, Shenoy ES. The impact of a reported penicillin allergy on surgical site infection risk. *Clin Infect Dis*. 2018;66(3):329-336. https://doi.org/10.1093/cid/cix794
6. Khan DA, Banerji A, Blumenthal KG, et al. Drug allergy: a 2022 practice parameter update. *J Allergy Clin Immunol*. 2022;150(6): 1333-1393. https://doi.org/10.1016/j.jaci.2022.08.028
7. Thomas PA, Kern DE, Hughes MT, Chen BY, eds. *Curriculum Development for Medical Education: A Six-Step Approach*. 3rd ed. Johns Hopkins University Press; 2016.
8. Kutzin J, Ng G. “Red in the face” interprofessional simulation case scenario. *MedEdPORTAL*. 2011;7:8408. https://doi.org/10.15766/mep_2374-8265.8408

Copyright © 2024 Plager and Cutrer This is an open-access publication distributed under the terms of the Creative Commons Attribution license.

9. Cavuoto Petrizzo M, Barilla-LaBarca ML, Lim YS, et al. Utilization of high-fidelity simulation to address challenges with the basic science immunology education of preclinical medical students. *BMC Med Educ*. 2019;19:352. https://doi.org/10.1186/s12909-019-1786-5

10. Blumenthal KG, Shenoy ES, Hurwitz S, Varughese CA, Hooper DC, Banerji A. Effect of a drug allergy educational program and antibiotic prescribing guideline on inpatient clinical providers' antibiotic prescribing knowledge. *J Allergy Clin Immunol Pract*. 2014;2(4):407-413. https://doi.org/10.1016/j.jaip.2014.02.003

11. Johnston EB, King C, Sloane PA, et al. Pediatric anaphylaxis in the operating room for anesthesia residents: a simulation study. *Paediatr Anaesth*. 2017;27(2):205-210. https://doi.org/10.1111/pan.13052

12. Weiner JJ, Eudy AM, Criscione-Schreiber LG. How well do rheumatology fellows manage acute infusion reactions? A pilot curricular intervention. *Arthritis Care Res (Hoboken)*. 2018;70(6):931-937. https://doi.org/10.1002/acr.23417

13. Kufel WD, Blaine BE, Ruehl R, Avery LM. Instruction and simulation to improve pharmacy students' knowledge and confidence regarding assessment of penicillin allergies. *Am J Pharm Educ*. 2022;86(3):8688. https://doi.org/10.5688/ajpe8688

14. Trubiano JA, Vogrin S, Chua KYL, et al. Development and validation of a penicillin allergy clinical decision rule. *JAMA Intern Med*. 2020;180(5):745-752. https://doi.org/10.1001/jamainternmed.2020.0403

15. Competencies for learners across the continuum. Vanderbilt University School of Medicine. Updated May 14, 2021. Accessed September 5, 2024. https://www.vanderbilt.edu/catalogs/kuali/som.php#/content/60da2185030bed556190f854

Received: May 31, 2023
Accepted: May 2, 2024
Published: September 27, 2024

Copyright © 2024 Plager and Cutrer This is an open-access publication distributed under the terms of the Creative Commons Attribution license.
